# Neurotherapeutic effects of Vutiglabridin as a Paraoxonase-2 modulator in preclinical models of Parkinson’s disease

**DOI:** 10.1186/s13024-025-00896-z

**Published:** 2025-10-17

**Authors:** Heeyoung An, Sora Kang, Jaejin Shin, Purum Kim, Sunpil Kim, Suyeol Im, Ji Hwan Kim, Keun Woo Lee, Dong Hwan Kim, Jung Hee Park, Min-Ho Park, Jaemin Lee, Sun Kyung Park, Kwang Pyo Kim, Hyeong Min Lee, Jae Ho Lee, Leo S. Choi, Hyun Ju Jeon, Suyeon Yellena Kim, In Young Hwang, Mridula Bhalla, Woojin Won, Hyung Soon Park, Sang-Ku Yoo, Byoung Dae Lee, C. Justin Lee, Youngmi Kim Pak

**Affiliations:** 1 Center for Cognition and Sociality, Institute for Basic Science (IBS), Daejeon, Republic of Korea; 2 Department of Neuroscience, Graduate School, Kyung Hee University, Seoul, Korea; 3 Glaceum Inc., Suwon, Korea; 4 Department of Biomedical Sciences, Graduate School, Kyung Hee University, Seoul, Korea; 5 Division of Life Science, Department of Bio & Medical Big Data, Research Institute of Natural Science, Gyeongsang National University, Jinju, Korea; 6 Division of Biotechnology, College of Environmental & Bioresources Sciences, Jeonbuk National University, Iksan, Korea; 7 Advanced Institute of Environment and Bioscience, College of Environmental & Bioresources Sciences, Jeonbuk National University, Iksan, Korea; 8 Department of New Biology, Daegu Gyeongbuk Institute of Science and Technology (DGIST), Daegu, Korea; 9 Department of Applied Chemistry, Institute of Natural Science, Global Center for Pharmaceutical Ingredient Materials, Kyung Hee University, Yongin, Korea; 10 Department of Biomedical Science and Technology, Kyung Hee Medical Science Research Institute, Kyung Hee University, Seoul, Korea; 11 Department of Physiology, School of Medicine, Biomedical Science Institute, Kyung Hee University, Seoul, Korea

**Keywords:** Paraoxonase, Mitochondria, Vutiglabridin, Parkinson’s disease, Therapeutics

## Abstract

**Background:**

Parkinson’s disease (PD) is the second most prevalent neurodegenerative disease characterized by motor impairment resulting from the degeneration of dopaminergic neurons in the substantia nigra, alongside α -synuclein (α-syn) accumulation, mitochondrial dysfunction, and oxidative stress. Recent studies on PD treatment have focused primarily on exploring oxidative stress and mitochondrial function as ways to restore dopamine release. Notably, previous studies have demonstrated that Paraoxonase 2 (PON2) plays a critical role in neuroprotection and neuroinflammation by reducing oxidative stress in striatal neurons and astrocytes.

**Methods:**

In this study, we investigated the potential therapeutic effect of a newly developed drug, Vutiglabridin, which is demonstrated to augment the activity of PON2 in the mouse model of PD. We assessed the impact of Vutiglabridin in a PD model induced by MPP^+^ treatment and overexpression of the A53T mutated α-syn. Furthermore, we administered Vutiglabridin subsequent to PON2 gene knockdown through PON2-shRNA overexpression to elucidate the interplay between PON2 and Vutiglabridin.

**Result:**

Vutiglabridin effectively crosses the blood-brain barrier (BBB) and maintains a presence in the brain for over 24 h, achieving concentrations up to 2.5 times higher than in the bloodstream. It successfully binds to PON2 in both its (R) and (S) forms. Vutiglabridin reversed mitochondrial dysfunction, reduced oxidative stress, improved motor functions, and protected dopaminergic neurons against MPP+-induced damage. Similarly, in α-syn A53T overexpressed PD models, it not only reduced astrocytic reactivity and microglia activation but also doubled the tyrosine hydroxylase positive neurons /dopa decarboxylase positive neurons (TH+/DDC+) ratio, signifying enhanced neuronal health. However, these positive outcomes were absent in PON2-knockdown mice, underscoring Vutiglabridin’s reliance on PON2 for its neuroprotective effects.

**Conclusion:**

These findings indicate that Vutiglabridin may serve as a promising therapeutic approach for reducing reactive oxygen species (ROS) levels by modulating PON2 activity in Parkinson’s diseases.

**Supplementary Information:**

The online version contains supplementary material available at 10.1186/s13024-025-00896-z.

## Introduction

Parkinson’s disease (PD) is the second most common neurodegenerative disease and also one of the fastest-growing neurodegenerative diseases globally in our aging society [1–3]. It is characterized by the selective loss of tyrosine hydroxylase (TH)-positive dopaminergic neurons in the substantia nigra pars compacta (SNpc) [3–5] and aggregation of alpha-synuclein (α-syn), known as Lewy bodies. While the precise etiology of PD remains undefined, ample evidence indicates the role of mitochondrial dysfunction and oxidative stress in its pathogenesis [6, 7]. The main mitochondrial defect in PD is associated with complex I (NADH-ubiquinone oxidoreduction) of the mitochondrial oxidative phosphorylation (OXPHOS); complex I activity was found to be decreased in post-mortem SNpc [8–10]. Dopaminergic neurons are particularly vulnerable to oxidative stress due to the presence of both dopamine and iron because dopamine is autoxidized into quinone form, and iron catalyzes the Fenton reaction, generating high amounts of superoxide radicals and hydrogen peroxide [11–13]. Mitochondrial dysfunction observed in PD patients further increases the generation of reactive oxygen species (ROS), including superoxide, which may trigger a cascade of events leading to the death of dopaminergic neurons [14].

Recent studies have elucidated the crucial role of astrocytes, as it is important for the inhibition of excessive ROS production from reactive astrocytes in PD pathology [15]. Inhibition of Monoamine oxidase B (MAO-B), located in the mitochondria of astrocytes, has been shown to significantly reduce ROS production, thereby attenuating the neurodegenerative process in PD [15, 16]. The previous studies highlight their findings in exacerbating PD symptoms through the production of gamma-aminobutyric acid (GABA) from astrocytes. The inhibition of MAO-B leads to a decrease in astrocytic GABA levels, which is pivotal in mitigating motor dysfunction, preserving TH + neurons in the SNpc, and reducing astrocyte and microglia hypertrophy observed in both the 1-methyl-4-phenyl-1,2,3,6-tetrahydropyridine (MPTP) and α-syn A53T overexpression PD models [15–17]. These results highlight the importance of targeting astrocytic ROS as a therapeutic strategy to alleviate PD symptoms and possibly halt disease progression. However, the effect of inhibiting astrocytic ROS in the PD pathology has not been directly tested.

During the progress of PD pathogenesis, increasing ROS in the astrocytes leads to hypertrophy of astrocytes, cytokine release, and mitochondria dysfunction. Meanwhile, mitochondria are a well-known source of ROS in physiological conditions, and ROS production is augmented and the cause of neuronal death in pathological conditions. Regulation of specific enzymes is the well-used path to suppress excessive ROS production from mitochondria.

Paraoxonase 2 (PON2) with lactonase enzyme activity is a ubiquitously expressed intracellular membrane protein with antioxidant properties that specifically reduce mitochondrial superoxide release [18–20]. PON2 is recognized as the earliest gene within the paraoxonase family that includes PON1, PON2, and PON3 [21], and stands out for its unique expression in the brain tissue of both humans and rodents [22, 23]. Very low levels of PON1 in the brain were detected [23], while PON2 exhibits a higher expression in the brain’s dopaminergic regions [23, 24], particularly in astrocytes more than neurons [25–27]. These findings lead us to focus on the therapeutic effect of Vutiglabridin through its interaction with PON2 in Parkinson’s Disease.

Since Parkinson’s disease is a neurodegenerative disorder, aging is a significant risk factor. The expression of PON2 has been reported to correlate with age and sex in the brains of non-human primates. PON2 levels increase with age up to the infant stage and then decrease at the juvenile stage, similar to the observations in mouse brain. The expression of PON2 in astrocytes is higher in female than male mice, rats, monkeys, and humans [28–30].

The risk of developing Parkinson’s disease is twice as high in men than in women, and the incidence of PD is 90% higher in males [31–33]. Lower PON2 levels in dopaminergic neurons in males may thus provide less defense against oxidative stress. These studies imply the association of PON2 expression in PD pathology.

PON2 exerts a neuroprotective role by reducing oxidative stress in striatal neurons and astrocytes. Cells from mice lacking PON2 were more susceptible to oxidants, especially in astrocytes than neurons, striatum than cerebellum [28]. It has been reported that PON2 also exhibits anti-inflammatory effects in macrophages [34]. These findings suggest that PON2 has a protective role toward oxidative stress and neuroinflammation which are closely related with PD etiopathology.

Vutiglabridin (HSG4112, hereinafter referred to as Vuti) is a novel small-molecule drug that increases the activity of PON2 in cells under stressed conditions [35]. Vuti is a chemically stable and potent derivative of Glabridin [36], a naturally occurring prenylated polyphenolic isoflavan with strong anti-oxidative and anti-inflammatory effects [37–39]. The safety of Vuti was verified through a successful Phase 1 clinical trial (NCT04732988, NCT04733001, NCT04703764), and Phase 2a trial (NCT06329141; A Study to Assess the Efficacy and Safety of Vutiglabridin in Early Parkinson’s Disease Patients) will be initiated to investigate the efficacy and safety of Vutiglabridin in early Parkinson’s disease.

In this study, we hypothesized that Vuti exerts an anti-Parkinson effect by interacting with PON2 and reducing ROS. Here, we found that Vuti reversed mitochondrial damage in neuronal cells and significantly ameliorated dopaminergic cell death and impaired motor behavior in MPTP and A53T PD mice models. The knockdown of PON2 abolished the effects of Vuti in PD models, suggesting that PON2 is required for Vuti’s mode of action. Therefore, Vuti emerges as a promising drug candidate for PD treatment by preventing dopaminergic cell death through its interaction with PON2.

## Results

### Vutiglabridin penetrates the blood-brain barrier in rodents

**Fig. 1:**
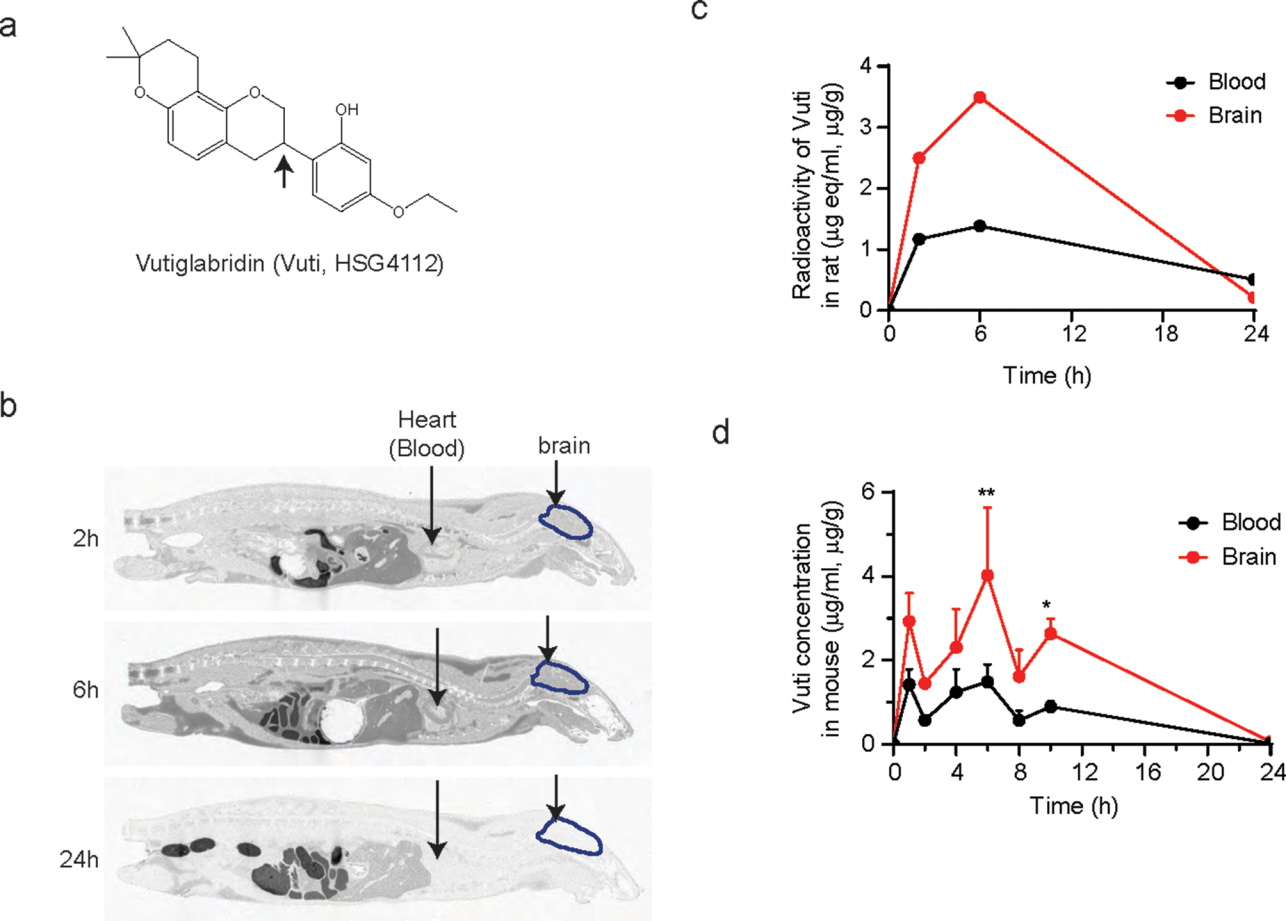
Vutiglabridin penetrates the brain. (**a**) Chemical structure of Vuti. The asterisk denotes stereocenter. (**b**) Whole-body autoradiograms shown from the central axis aspect at 2 h, 6 h, and 24 h after a single oral administration of 10 mg/kg of ^14^C-labeled Vuti to a fasted male SD rat. Arrows show the site of the brain and heart (blood). (**c**) Comparative radioactivity of ^14^C-labeled Vuti in the brain and blood (*n* = 1 per group per timepoint). (**d**) The concentration of Vuti in the plasma and brain of male C57BL/6J mice after a single oral administration of 50 mg/kg was measured via LC-MS/MS analysis. The data are plotted as mean ± SEM (*n* = 3 per time point at 0, 1, 2, 4, 6, 8, 10, and 24 h). *p < 0.05, **p < 0.01 vs. Blood. *p* values are from a two-way ANOVA followed by Sidak’s test

Firstly, we hypothesized that because of its hydrophobicity and small molecular weight, Vuti would cross the BBB, which is critical for the potential direct engagement of the dopaminergic neurons. As shown by the chemical structure in Fig. 1a, Vuti is a highly hydrophobic compound with an octanol-water partition coefficient (log D) greater than 3.69 (Supplemental Figure S1a). To assess the ability of Vuti to cross the BBB, we performed an in vitro permeability test, determining whether Vuti is a substrate for P-glycoprotein (P-gp, or multidrug resistance protein 1, MDR1). The efflux ratio of Vuti was measured using LC-MS/MS in a standard set of cell monolayers (MDCKII, MDCKII-hMDR1, and Caco-2 cells) [40], both with and without a P-gp inhibitor. Under all tested conditions, Vuti showed a low efflux ratio, suggesting it is not a substrate for P-gp (Supplemental Figure S1b). To investigate the brain distribution of Vuti, a standard quantitative whole-body autoradiography (QWBA) analysis was then performed in Sprague-Dawley (SD) rats [41] using ^14^C-Vuti synthesized as described in Supplemental Figure S1c. After a single administration of 10 mg/kg of ^14^C-Vuti, the maximum plasma concentration was reached at 6 h (T_max_) (Supplemental Figure S1d). Subsequently, whole-body sagittal sections were prepared from another set of SD rats, each sacrificed at 2, 6, and 24 h time points, to quantify the spatial and temporal distribution of ^14^C-Vuti. The whole-body autoradiogram showed that the ^14^C-Vuti compound was distributed in most organs of the body except for bone and seminal vesicle (Fig. 1b, Supplemental Table S1). Notably, ^14^C-Vuti was successfully distributed to the brain at a level 2.5-fold higher compared to those in the plasma at the T_max_ of 6 h (Fig. 1c). We further evaluated the brain distribution of Vuti in C57BL/6J mice. The concentration in the brain after a single administration of 50 mg/kg of Vuti was 2.7-fold higher than that in plasma at the T_max_ of 6 h (4.03 µg/g in brain vs. 1.49 µg/ml in plasma) (Fig. 1d). Considering the total area under the curve exposure (AUC_24h_) (42.5 µg∙h/g in brain vs. 16.1 µg∙h/ml in plasma), the concentration in the brain was 2.6-fold higher than in plasma. These results consistently confirm that Vuti successfully penetrates the BBB and achieves substantial brain distribution.

### Vutiglabridin protects against mitochondrial damage induced by MPP^+^

**Fig. 2:**
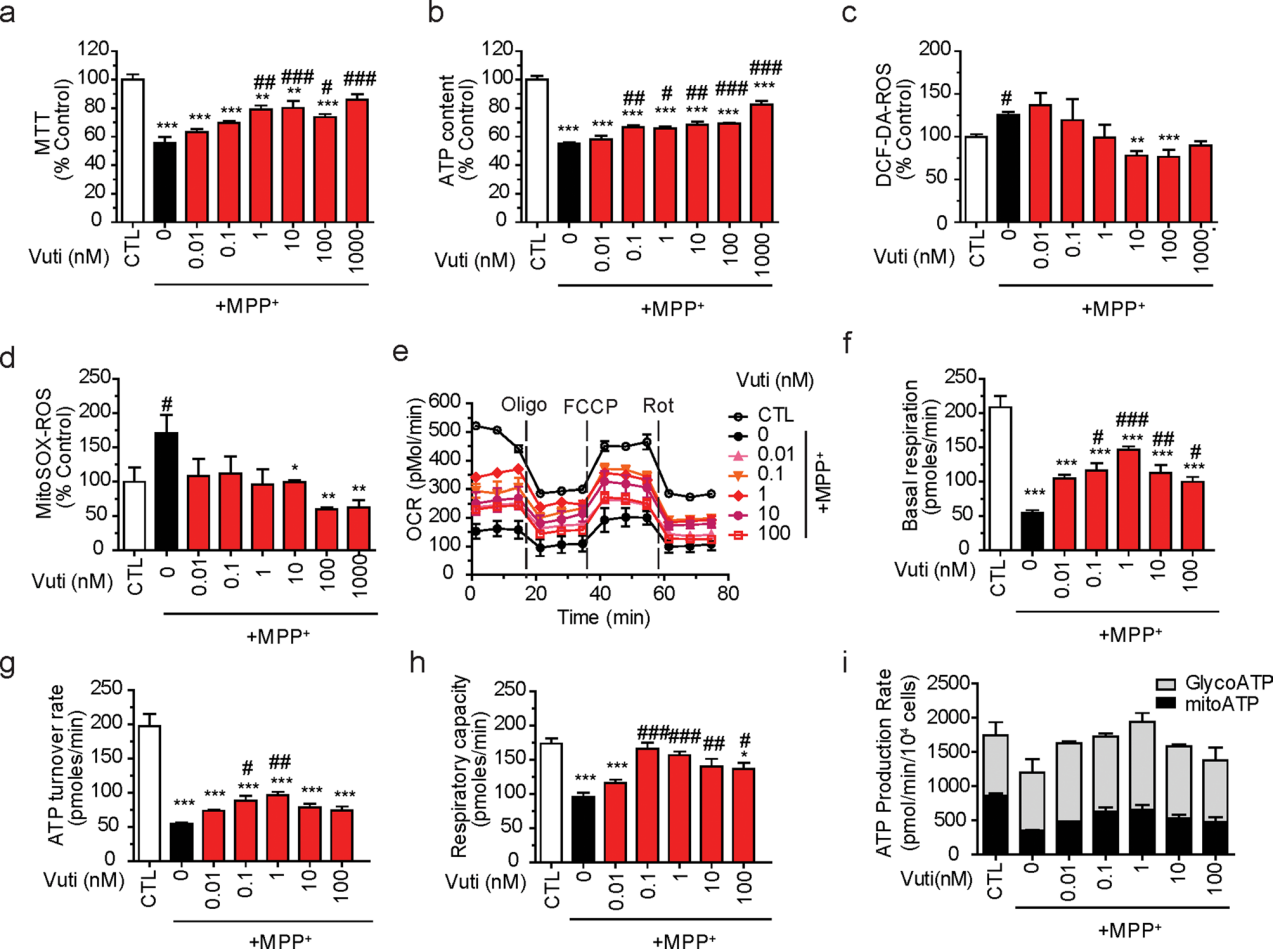
Dose-dependent effects of vutiglabridin on MPP^+^-induced cellular and mitochondrial damage in SH-SY5Y neuronal cells. SH-SY5Y cells were placed in a 96-well plate and incubated with 1 mM MPP^+^ for 24 h and then were post-treated with Vuti (0 ~ 1 µM) for 24 h. The mitochondrial activity was analyzed. (**a**) MTT as NADH dehydrogenase activity of OXPHOS complex 1. (**b**) Intracellular ATP content. (**c**) DCF-DA-based total reactive oxygen species (ROS) generation. (**d**) MitoSox-based mitochondrial superoxide generation. (**e** ~ **g**) SH-SY5Y cells were placed in an XF-24 microplate and incubated with 1mM MPP^+^ for 20 h and then were post-treated with Vuti (1 ~ 100 nM) for 24 h. The oxygen consumption rates (OCR) were analyzed using a Seahorse XF-24 analyzer. (**e**) OCR profile. Oligomycin (Oligo), FCCP, and rotenone (Rot) were consecutively injected to obtain mitochondrial respiratory capacities. (**f**) Basal respiration OCR. (**g**) ATP turnover rate (basal OCR – oligomycin-inhibited OCR). (**h**) Total respiratory capacity (FCCP-induced OCR). (**i**) Using ECAR and OCR profiles, ATP production rates through glycolysis (GlycoATP, grey bar) or mitochondria (mitoATP, black bar) were calculated. Mitochondrial activity values are reported as a percentage of the control (CTL). The data are plotted as the mean ± SEM (*n* = 3). *p < 0.05, **p < 0.01, ***p < 0.001 vs. CTL (white bar); ^#^*p* < 0.05, ^##^*p* < 0.01, ^###^*p* < 0.001 vs. MPP^+^-treated control (Black bar). *p* values are from a one-way ANOVA followed by Tukey’s test

To investigate whether Vuti mediates its protective effect against mitochondrial damage, potentially by modulating PON2, using MPP^+^ toxin as a PD model (where MPP^+^ acts as a mitochondrial complex I inhibitor). First, SH-SY5Y human neuroblastoma cells were exposed to 1 mM MPP^+^ for 24 h, followed by 24 h post-treatment of Vuti. Vuti significantly reversed MPP^+^-induced mitochondrial damage in a dose-dependent manner. This was evident in the restoration of NADH dehydrogenase complex 1 activity (Fig. 2a), intracellular ATP content (Fig. 2b), DCF-DA-mediated cellular ROS (Fig. 2c), and MitoSOX-mediated mitochondrial superoxide (Fig. 2d). When oxygen consumption rate (OCR), an important physiological indicator of mitochondrial activity [42], was determined, Vuti dose-dependently and significantly rescued MPP^+^-induced reduction in basal respiration, ATP turnover rate, and maximum respiratory capacity (Fig. 2e–h). Vuti also normalized ATP production rates through both glycolysis (GlycoATP) and mitochondria (mitoATP) (Fig. 2i). Given the significant restoration of MPP^+^-induced damage was observed at 1 nM of Vuti, this concentration was subsequently used to further investigate the neuroprotective effects of Vuti. These results demonstrate the efficacy of Vuti in ameliorating all aspects of neurotoxin-induced mitochondrial dysfunction in neuronal cells.

### Vutiglabridin protects dopaminergic neurons and motor behavior in MPTP-injected mice

**Fig. 3:**
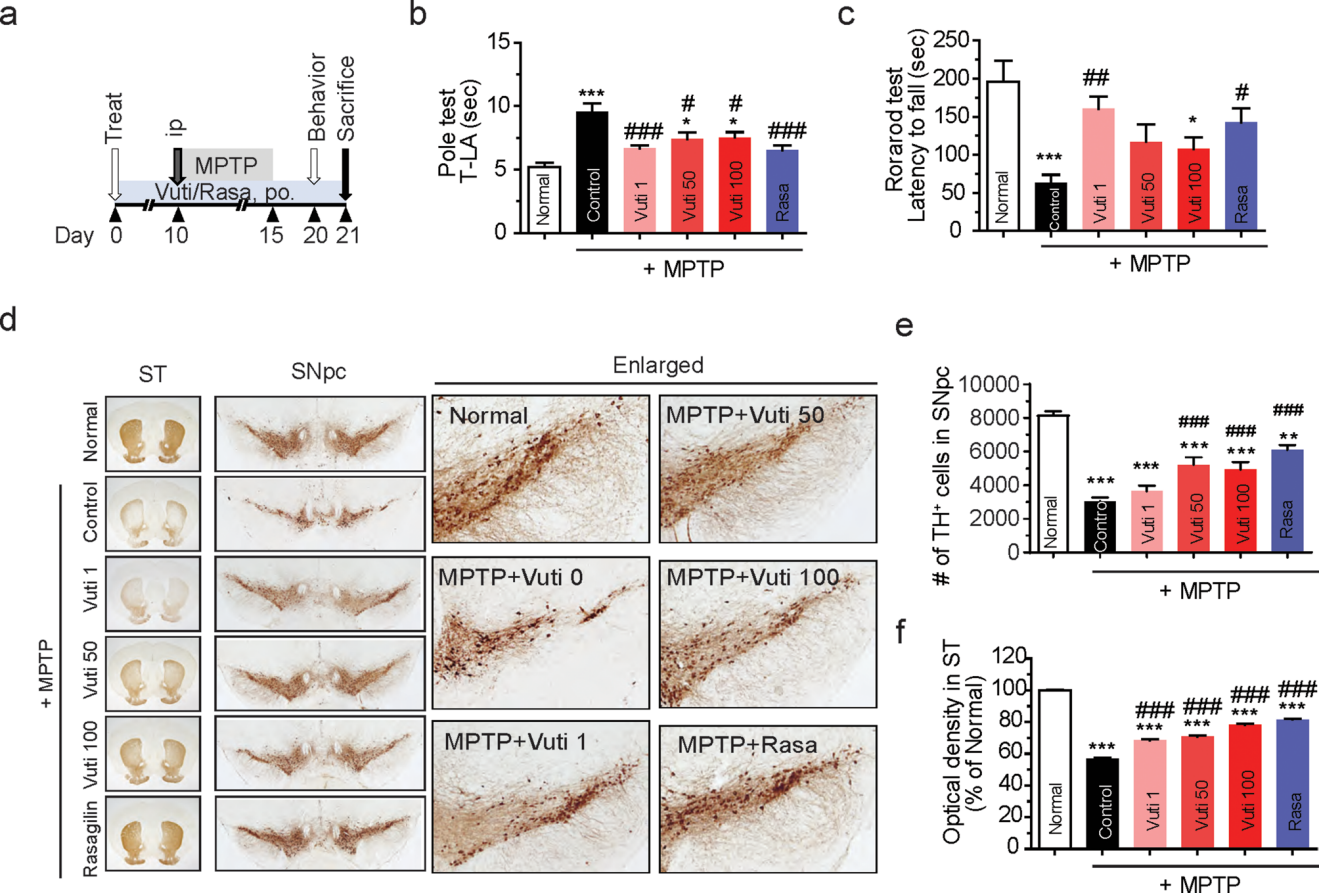
Vutiglabridin attenuates motor impairment and protects dopaminergic neurons in MPTP-injected PD mice. (**a**) Scheme of the experimental design. C57BL/6 mice (male 8–10 weeks old, *n* = 5 per group) were orally administered with Vuti (1, 50, or 100 mg/kg/day) or rasagiline (Rasa, 0.1 mg/kg/day) for 21 consecutive days. All groups except for the normal control group were injected with MPTP intraperitoneally at 30 mg/kg/day for five days starting on day 10. On day 20, the rotarod and pole tests were performed. On day 21, mice were sacrificed, and dopaminergic neurons were visualized via tyrosine hydroxylase (TH) immunohistochemistry. (**b**) Latency time in the rotarod test. (**c**) Latency time to arrive at the floor (T-LA) in the pole test. (**d**) Representative photomicrographs of the SNpc and the striatum (ST). (**e**) Stereological count of the number of TH-immunopositive neurons in SNpc. (**f**) Optical density in the ST. Th e data are plotted as the mean ± SEM (*n* = 5). *p < 0.05, **p < 0.01, ***p < 0.001 vs. vehicle-only group; ^#^*p* < 0.05, ^###^*p* < 0.001 vs. vehicle-only group injected with MPTP. *p* values are from a one-way ANOVA followed by Tukey’s test

Next, we investigated whether the in vitro effects of Vuti could be reproduced in the MPTP toxin in vivo model of PD. A sub-acute PD model in C57BL/6J mice was established by injecting MPTP (30 mg/kg/day, *i.p.*) for 5 days, as described in the experimental scheme (Fig. 3a). Various doses of Vuti (0, 1, 50, and 100 mg/kg/day) were orally administered for 10 days before MPTP injection and continued for a total of 3 weeks. Rasagiline (Rasa, 0.1 mg/kg), an irreversible inhibitor of MAO-B, was used as a positive reference drug. Injection of MPTP significantly increased latency time to reach the floor (T-LA) in the pole test (9.6 ± 2.1 s vs. 5.2 ± 0.9 s, *p* < 0.001) (Fig. 3b) and decreased latency to fall in the rotarod test (63.5 ± 34.5 s vs. 196.4 ± 77.3 s, *p* < 0.001) (Fig. 3c). Treatment with both Vuti and rasagiline significantly normalized the T-LA in the pole test and the time of latency to fall in the rotarod test. Immunohistochemical staining showed significantly more TH-positive cells in the SNpc and TH-positive fibers in the ST, compared to the vehicle-only (Normal) group (Fig. 3d). Vuti dose-dependently increased the number of TH-positive cells in the SNpc and fiber density in the ST (*p* < 0.001 vs. control) to levels comparable to those of rasagiline (Fig. 3e–f). These results consistent with the findings in SH-SY5Ycells, suggest that Vuti effectively ameliorates MPTP-induced damage in PD mice. No discernible difference in neuroprotection was observed between 50 and 100 mg/kg of Vuti, establishing 50 mg/kg as the maximum efficacy dose for subsequent in vivo studies.

### Vutiglabridin binds to PON2 in vitro and in vivo

**Fig. 4:**
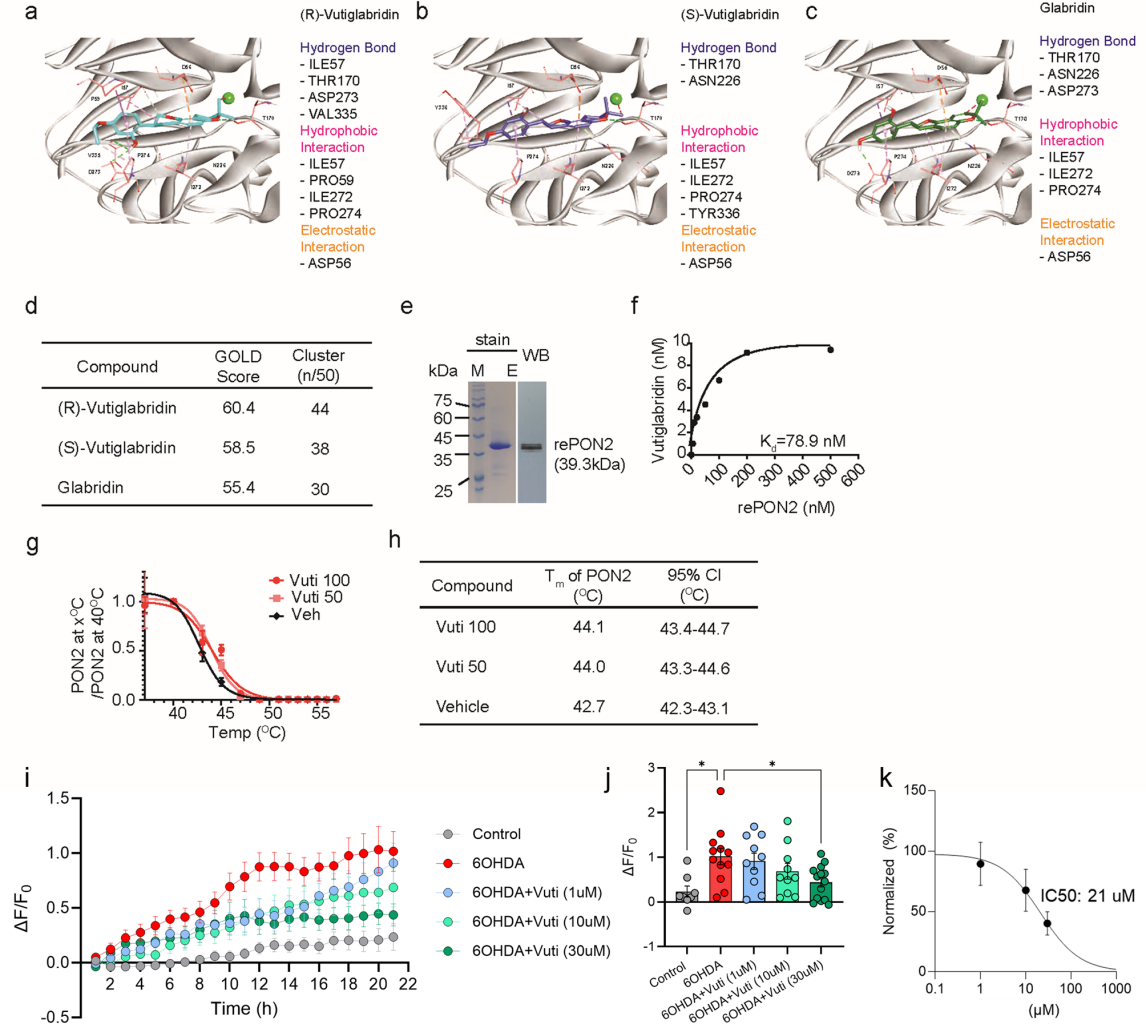
De novo 3D structure of PON2 and binding interaction with vutiglabridin. (**a** ~ **c**) The binding site of PON2 and the molecular docking simulation of (R)-Vut (A), (S)-Vuti, (B), and glabridin (**c**) to PON2, showing sites of hydrogen bonding, hydrophobic interaction, and electrostatic interaction. (**d**) Scoring of molecular docking by GOLD fitness score and cluster match analysis. (**e**) Coomassie blue staining of SDS-PAGE and Western blot of final purified recombinant PON2 (rePON2). (**f**) MS-based binding assay. rePON2 at various concentrations was incubated with 10 nM of Vuti for 30 min, and the quantity of rePON2-bound Vuti was analyzed by LC-MS/MS. The dissociation constant (K_d_) was 78 nM. Data is plotted as mean ± SEM (*n* = 3; the error bar is not visible because the error range is negligible). (**g**) Cellular thermal shift assay (CESTA^®^). Melting and thermal shift curves are shown for PON2 in brain tissue from mice treated with Vuti 100 mg/kg (Vuti 100), 50 mg/kg (Vuti 50), or vehicle (Veh). PON2 band intensity at x℃ was normalized to PON2 band intensity at 40℃. (**h**) Compiled T_m_ values for PON2 from (**g**) including the 95% confidence interval (CI) values (*n* = 6). (**i** ~ **j**) ROS levels after treatment with 6-OHDA in primary cultured astrocytes expressing the oROS-G sensor, in a dose-dependent manner of Vuti (ranging from 1 µM to 30 µM). (**k**) The normalized IC_50_ of Vuti is 21 µM, determined by oROS-G sensor imaging

To evaluate the potential binding interaction of Vuti with PON2, we constructed a *de novo* three-dimensional (3D) in silico model of PON2 based on a homology modeling with PON1, the paraoxonase family members. This approach was chosen because the 3D structure of PON2 remains unknown, whereas that of PON1 has been reported, and PON1 showed 61.7% sequence identity and 79.2% sequence similarity with PON2 (Supplemental Figure S2). The newly constructed 3D protein structure of PON2 revealed the docking site for Vuti. The structural stability of the constructed PON2 was verified through molecular dynamic simulation, which showed stable maintenance for 10 ns and adequate interatomic collisions, as evidenced by the Ramachandran plot. Molecular docking simulation showed that both (R) and (S) forms of Vuti established hydrophobic and non-hydrophobic (polar and electrostatic) interactions with PON2 (Fig. 4a **~ **d); they had cluster matches of 44 and 38 out of 50, and Genetic Optimization for Ligand Docking (GOLD) fitness score of 60.4 and 58.5, respectively. Glabridin also had similar binding interactions with PON2 but with lower scores in both cluster match and GOLD fitness, suggesting that Vuti binds to PON2 more effectively than glabridin.

To verify the binding of Vuti to PON2, mass spectrometry-based binding assays [40, 41] were performed using stable and soluble recombinant human PON2 (rePON2) with C-terminal His-tag [43–45]. In this in-tube assay, various concentrations of rePON2 were incubated with 10 nM of Vuti, and the amount of Vuti bound to rePON2-bound was analyzed using LC-MS/MS. The amount of Vuti bound to rePON2 increased with increasing rePON2 concentration, and the calculated dissociation constant (K_d_) was 78 nM (Fig. 4e–f), indicating that Vuti binds to PON2 with relatively high affinity in the nanomolar range. Next, we determined PON2 protein thermal stability by CESTA^®^ thermal shift assay. From this assay, melting and shift curves were generated for brain tissue from mice treated with 100 mg/kg Vuti (Vuti 100), 50 mg/kg Vuti (Vuti 50), or vehicle (Veh) for five consecutive days before the sacrifice. Heat-challenge at 12 different temperatures (37 to 56℃) showed that both Vuti 100 and Vuti 50 treatment significantly enhanced the thermal stability of PON2 compared to the vehicle control group (Fig. 4g). It was confirmed that Vuti administration increased the T_m_ of PON2 by approximately 1.3℃ (Vuti 50) to 1.4℃ (Vuti 100) than that of the vehicle group (Fig. 4h). Collectively, these data suggest that Vuti binds directly to PON2.

Additionally, to investigate whether Vuti reduces ROS in primary cultured astrocytes, we treated the cells with Vuti following 6-OHDA treatment to simulate the Parkinson’s disease condition in vitro. We observed a dose-dependent reduction in ROS levels, with higher concentrations of Vuti (ranging from 1 µM to 30 µM) leading to progressively lower ROS levels in the 6-OHDA-treated astrocytes (Fig. 4i–k). Notably, 30 µM of Vuti significantly reduced ROS levels in these astrocytes. Based on these results, along with evidence that Vuti binds to PON2, we propose that Vuti reduces ROS through the activation of PON2 in astrocytes.

### Knockdown of PON2 abrogates the neuroprotective effects of vutiglabridin in MPP^+^-treated cells

**Fig. 5:**
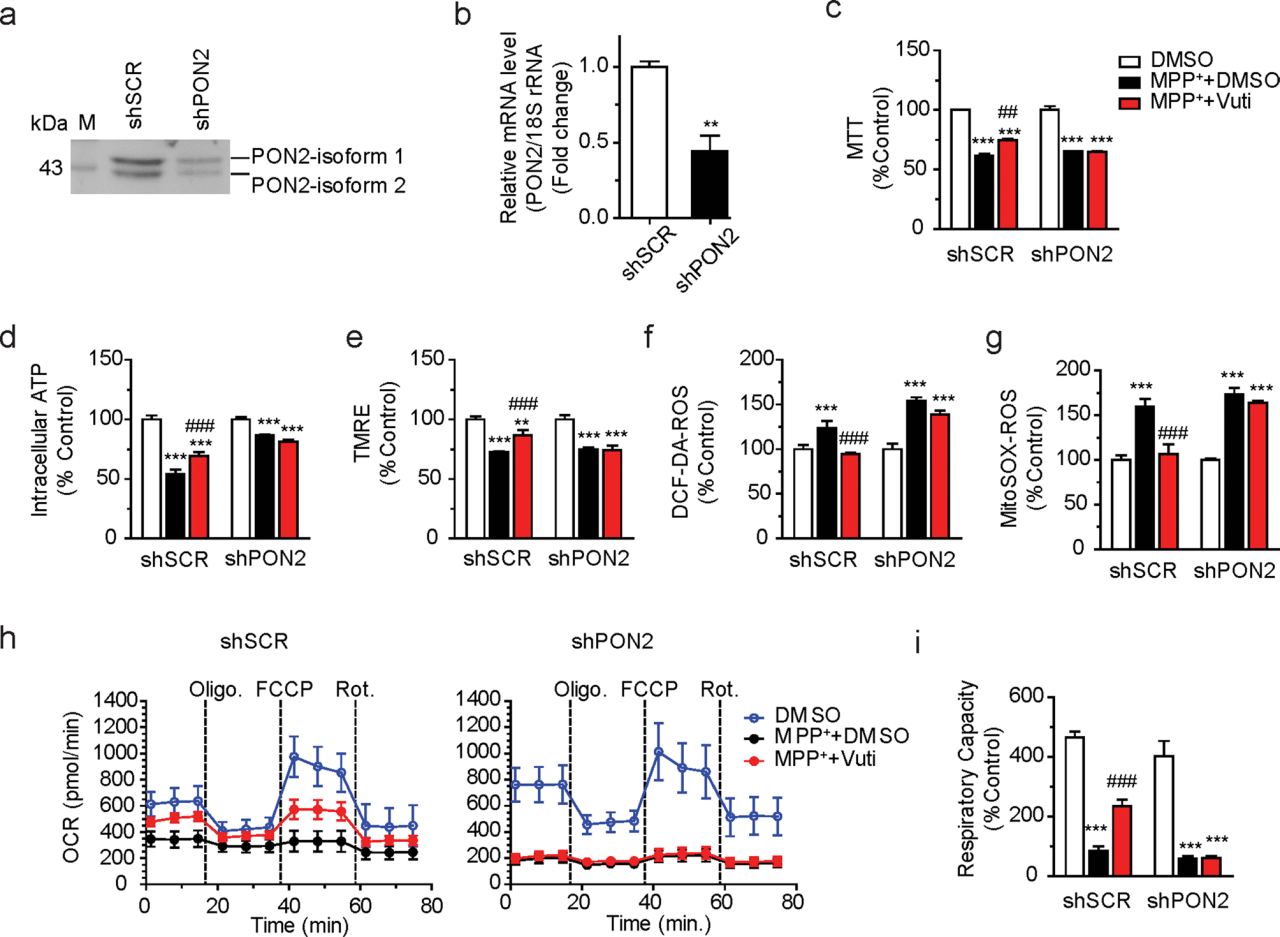
Vutiglabridin restores MPP^+^-induced damage in neuronal cells via PON2. PON2 knockdown (KD) SH-SY5Y cells were established by infecting lenti-shPON2 viral particles. Verification of PON2 KD by (**a**) Western blot and (**b**) real-time PCR. The values are reported in comparison to the control (shSCR). (**c** ~ **g**) shPON2-SH-SY5Y (shPON2) cells were placed in a 96-well plate and incubated with 1 mM MPP^+^ for 20 h, and then treated with 1 nM Vuti for 24 h, after which the mitochondrial activity was analyzed. (**c**) MTT as NADH dehydrogenase activity of OXPHOS complex 1. (**d**) Intracellular ATP content. (**e**) TMRE-mediated mitochondrial membrane potential. (**f**) DCF-DA-based total ROS generation. (**g**) MitoSOX-based mitochondrial superoxide generation. All values are reported as a percentage of the Control. The data are plotted as the mean ± SEM (*n* = 6). Cells in the XF-24 microplate were incubated with 1 mM MPP^+^ for 20 h and then treated with 1 nM Vuti for 24 h. OCR was analyzed using a Seahorse XF-24 analyzer. Oligomycin, FCCP, and rotenone were consecutively injected to obtain mitochondrial respiratory capacities. (**h**) OCR profiles of shSCR control cells and shPON2 cells. (**i**) Total respiratory capacity (FCCP-induced OCR). The data are plotted as the mean ± SEM (*n* = 3). *p < 0.05, **p < 0.01, ***p < 0.001 vs. Control (DMSO, white bar); ^#^*p* < 0.05, ^##^*p* < 0.01, ^###^*p* < 0.001 vs. MPP^+^ + DMSO (black bar). *p* values are from a one-way ANOVA followed by Tukey’s test

To assess whether PON2 is essential for the neuroprotective effects of Vuti against MPP^+^-induced damage in SH-SY5Y cells, we established a PON2 knockdown (KD) cells by infecting SH-SY5Y cells with lentivirus-based PON2 shRNA (shPON2). Western blot and real-time RT-qPCR analyses confirmed that the expression of PON2 protein (both isoforms) and mRNA was reduced by approximately 60% (Fig. 5a and b) in the shPON2-infected PON2-KD cells (shPON2 cells). In cells infected with control scrambled shRNA (shSCR cells), Vuti significantly restored MPP^+^-induced mitochondrial damages, consistent with the results in Fig. 2. However, in shPON2 infected cells, the effects of Vuti on mitochondria function were considerably reduced or almost completely abolished, as measured by MTT (NADH dehydrogenase activity), intracellular ATP content, TMRE-membrane potential, DCF-DA-ROS, and MitoSOX-ROS (Fig. 5c–g). In addition, OCR measurement showed that Vuti significantly restored MPP^+^-induced damages in basal respiration, ATP turnover rate, and maximum respiratory capacity to control levels in shSCR cells, whereas Vuti failed to restore them in shPON2 cells (Fig. 5h and i). Of note, in the shPON2 cells compared to shSCR cells, MPP^+^ treatment further increased both cellular and mitochondrial ROS (Fig. 5f and g) and also completely reduced the overall OCR profile (Fig. 5h). This suggests that shPON2 cells may be more susceptible to MPP^+^-induced mitochondrial damage. These results indicate that PON2 is indispensable for the neuroprotective effects of Vuti against mitochondrial toxins.

### No neuroprotective effects of vutiglabridin in PON2-knockdown mice

**Fig. 6:**
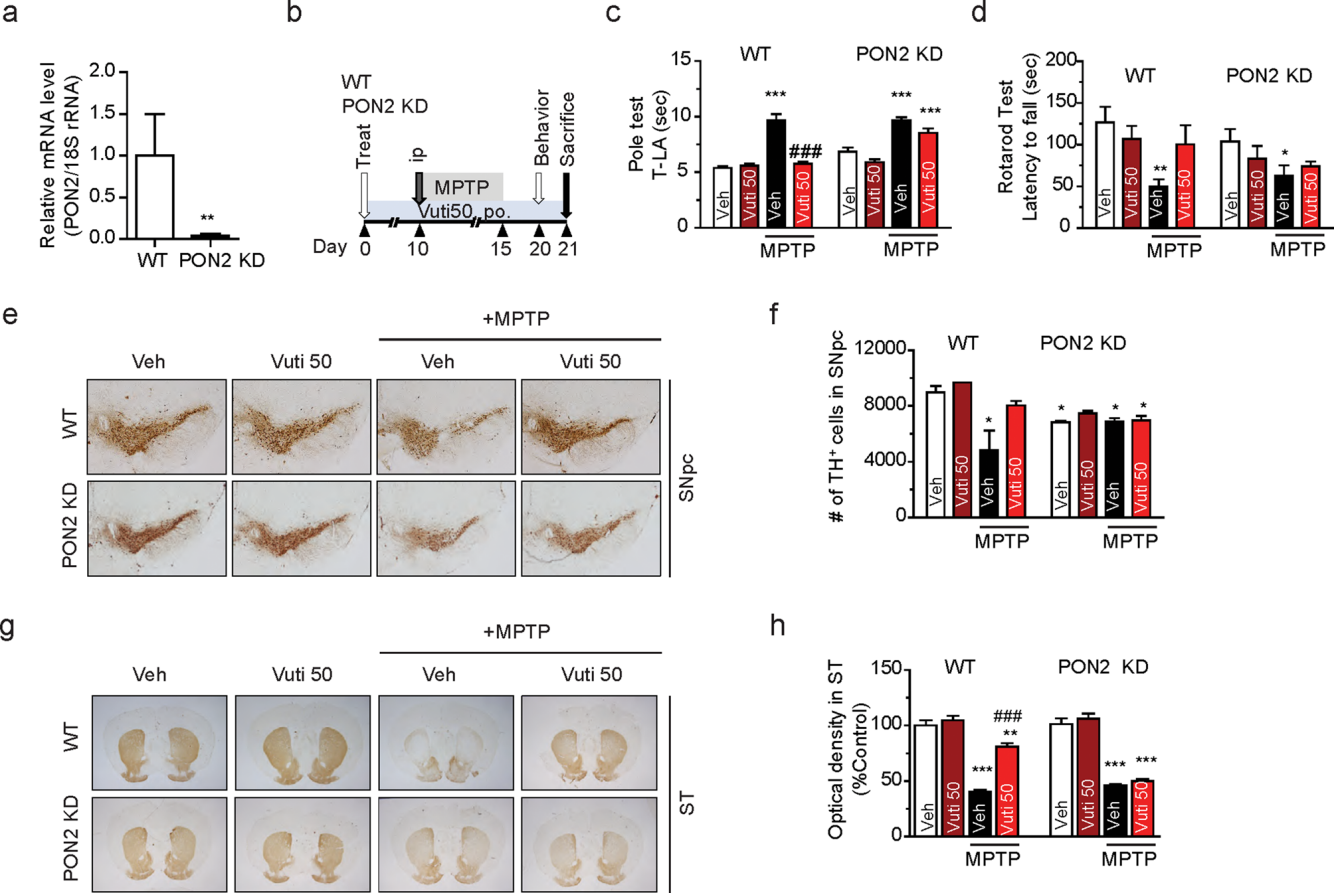
Vutiglabridin protects MPP^+^-induced damage in mice via PON2. (**a**) Realtime RT-PCR of PON2 mRNA level in the total brain homogenates of WT and PON2 KD mice. The data are plotted as the mean ± SD (*n* = 5 ~ 8). **p < 0.01 vs. WT, from a student’s t-test. (**b**) Scheme of the experimental design. Male wild-type C57BL/6 mice (WT) and PON2-knockdown mice (KD) of 8–10 weeks of age were orally administered with 0 or 50 mg/kg of Vuti (Vuti 0 and Vuti 50) for 21 consecutive days (*n* = 5 ~ 8). All groups except for the vehicle-only treated group were injected with MPTP intraperitoneally at 30 mg/kg for five days starting on day 10. All compounds were administered daily. On day 20, the rotarod and pole tests were performed. On day 21, mice were sacrificed, and dopaminergic neurons were visualized via TH immunohistochemistry. (**c**) Latency time (sec) to arrive at the floor (T-LA) in the pole test. (**d**) Latency time (sec) in the rotarod test. (**e**) Representative photomicrographs of the SNpc. (**f**) Stereological count of the number of TH + neurons in SNpc. (**g**) Representative photomicrographs of the ST. (**h**) Optical density in the ST. The data are plotted as the mean ± SEM (*n* = 5 ~ 8). The number of animals for each group is specified in the methods. *p < 0.05, ***p < 0.001 vs. Vehicle group (Vuti 0 without MPTP); ^#^*p* < 0.05, ^###^*p* < 0.001 vs. MPTP + Vehicle group (Vuti 0). *p* values are from a one-way ANOVA followed by Tukey’s test

To determine if PON2 mediates the neuroprotective effects of Vuti in an in vivo model, we used PON2 knockdown (PON2-KD) mice in a subacute PD model induced by MPTP injection. These PON2-KD mice were generated using a gene trap vector with an insertional mutation in the second intron of PON2 [46]. The PON2-KD model has been reported to have a slight leakage (< 10%) of PON2 in the whole body [46], but PON2 mRNA levels in the brain were almost completely diminished (Fig. 6a). Administrating 50 mg/kg Vuti (Vuti 50) for three weeks significantly attenuated MPTP-induced damage to motor behaviors and dopaminergic neurons in wild-type mice (Figs. 3 and 6), whereas these neuroprotective effects were absent in PON2-KD mice (Fig. 6b–h). Furthermore, a control group of non-MPTP-injected wild-type mice treated with 50 mg/kg Vuti showed no changes in motor behavior or dopaminergic neuronal cell count and fiber density compared to the vehicle-only treated group. This indicates that Vuti itself does not induce any behavioral or neuronal damage in wild-type mice and that the efficacy observed in the MPTP model is strictly due to the effects of Vuti against MPTP injection. These results demonstrate that the most significant protective effects of Vuti in the MPTP model are observed at a dose of 50 mg/kg, with PON2 mediating these neuroprotective effects.

### Vutiglabridin mitigates α-syn pathology and protects dopaminergic neurons in the AAV-A53T PD model

The effect of Vuti on α-syn pathology was validated using a mouse model injected with AAV-α-syn A53T mutant (AAV-A53T) into SNpc. While Vuti was pre-treated before MPTP injection in the MPTP model study, it was post-treated after AAV-A53T injection in the SNpc for inducing AAV-A53T model (A53T group) to assess its potential as a disease-modifying drug. The previous study showed the amelioration of motor locomotion, GFAP hypertrophy, increase of TH+/DDC + cell ratio, and tonic GABA decrease by the post-treated inhibitor of MAO-B [15, 16]. In the GABA synthesis pathway, MAO-B converts the N-acetyl putrescine to N-acetyl-γ-aminobutyraldehyde and produces H_2_O_2_ as a byproduct [15–17]. Despite this, neither MAO-A nor MAO-B is effectively inhibited by Vuti, resulting in minimal impact on reducing reactive oxygen species (ROS) levels or improving symptoms in PD model mice (Supplemental Figure S3). Consequently, Vuti’s effects on behavior and ex vivo analyses were further investigated, independent of MAO enzyme involvement, leading to the conclusion that its therapeutic potential is largely unrelated to MAO activity.

**Fig. 7:**
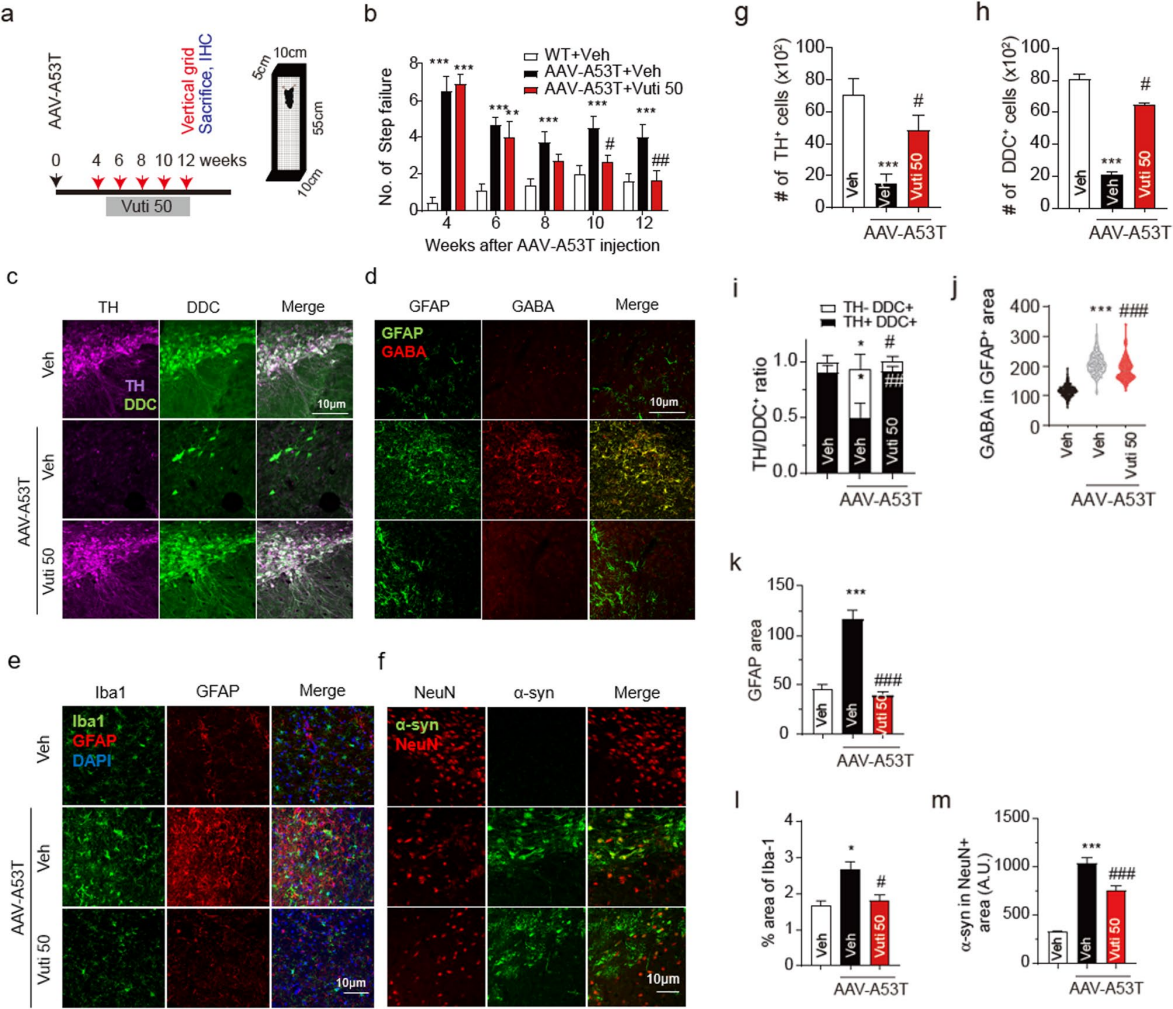
Vutiglabridin restores the loss of dopaminergic neurons in SNpc and motor impairment of the A53T mouse model. (**a**) Experimental procedure of behavior test. (**b**) Step failure for vertical grid of WT + Veh (*n* = 10), A53T + Veh (*n* = 10) and A53T + Vuti 50 (*n* = 10) group. (**c**) Immunofluorescence images of TH (magenta), and DDC (green) in the WT + Veh, A53T + Veh, and A53T + Vuti 50 groups. Scale bar = 50 μm. (**d**) Immunofluorescence images of GFAP (green), and GABA (red) in the WT + Veh, A53T + Veh, and A53T + Vuti 50 groups. Scale bar = 50 μm. (**e**) Immunofluorescence images of Iba-1 (green), GFAP (red), and DAPI (blue). Scale bar = 10 μm. (**f**) Immunofluorescence images of α-syn (green), NeuN (red), and DAPI (blue). Scale bar = 10 μm. (**g**) Comparison of the number of TH + cells in the WT + Veh (white bar, *n* = 3), A53T + Veh (black bar, *n* = 4), and A53T + Vuti 50 group (red bar, *n* = 4). (**h**) Comparison of number of DDC + cells. (**i**) Comparison of TH+/DDC + ratio in the WT + Veh (white bar, *n* = 8), A53T + Veh (black bar, *n* = 8), and A53T + Vuti 50 group (red bar, *n* = 6). (**j**) Comparison of GABA intensity in GFAP + cells in the WT + Veh (white bar, *n* = 3), A53T + Veh (black bar, *n* = 4), and A53T + Vuti 50 group (red bar, *n* = 4). (**k**) Comparison of averaged GFAP-positive area. (**l**) Comparison of averaged percent area of Iba-1 in the image window. (**m**) Comparison of the averaged α-syn-positive area in NeuN-positive area Data represents Mean ± SEM. *, *p* < 0.05; **, *p* < 0.01; ***, *p* < 0.001; vs. WT + veh. #, *p* < 0.05; ##, *p* < 0.01 vs. A53T + veh. p-values are from a one-way ANOVA followed by Tukey’s test

To investigate whether the protective effect of Vuti is observed in the genetic PD model, the AAV-A53T was bilaterally injected into the SNpc, and at four weeks post-injection, the manifestation of PD motor symptoms was confirmed by a vertical grid test. Starting from week five, post-AAV-A53T injection, Vuti was administered at a dose of 50 mg/kg for seven weeks, a dosage previously confirmed to be effective in the MPTP model. Throughout this period, the vertical grid test was conducted every two weeks to assess the effects. (Fig. 7a). The results showed a gradual improvement in motor behavior in the Vuti group compared to the vehicle group. Specifically, statistically significant improvements in step failure measures were observed within 5 to 7 weeks of Vuti administration (at weeks 10–12) (Fig. 7b). Tissue analysis at 12 weeks after AAV-A53T injection revealed a significant increase in TH-positive cells in the Vuti group compared to the vehicle group (Fig. 7c and g). In addition, DDC-positive cells were significantly increased (Fig. 7c and h), indicating the presence of dormant dopaminergic neurons. This suggests that surviving dopaminergic neurons can still be detected even among non-TH + cells. DDC + neurons are capable of functioning as normal dopaminergic neurons once stressors like α-syn and ROS are removed [15]. Vuti not only increased the number of TH + and DDC + cells but also alleviated the TH-/DDC + dormant dopaminergic neuron, thereby reactivating dormant dopaminergic neurons and improving motor function (Fig. 7i). Meanwhile, Vuti reduced inflammation by reducing GFAP, a marker for reactive astrocytes. This reduction in inflammation also led to a decrease in the amount of GABA in astrocytes. (Figure 7d and j).

Under the chronic influence of toxins such as α-syn and ROS, the glial cells become chronically activated and have difficulty eliminating α-syn. As a result, they are unable to perform their own functions and maintain dopaminergic neuron homeostasis. Vuti effectively reduced inflammation in glial cells, including astrocytes and microglia, leading to the normalization in the levels of GFAP and Iba1 levels (Fig. 7e and k–l). Given that normalized glial cell functions promote efficient removal of aggregated α-syn, Vuti administration resulted in decreased intracellular α-syn levels (Fig. 7f and m). In summary, Vuti significantly attenuated glial cell inflammation in the AAV-α-syn A53T model, protecting dopaminergic neurons by effectively removing α-syn.

Moreover, based on the PON2 activity assay established in our previous study, we observed that Vuti modulates PON2 activity (Supplemental Figure S4c). Specifically, while the PON2 protein level remained unchanged after Vuti treatment in both the A53T mouse model and the WT + Vuti group (Supplemental Figure S4 a and b). In addition, both esterase and lactonase activities, which were decreased following A53T virus exposure, were subsequently increased upon Vuti treatment in primary cultured astrocytes. Above all, these findings suggest that Vuti directly binds PON2 and regulates PON2 activity without altering its expression.

### Neurotoxicity assessment of vutiglabridin by in vitro safety screening and FOB analysis

The overall safety of Vuti has been already established through human phase 1 clinical trial (NCT04732988, NCT04733001, NCT04703764). However, for its potential repositioning as a central nervous system (CNS)-targeting drug candidate, we performed a comprehensive neurotoxicity assessment of Vuti through in vitro safety screening. Interactions of Vuti were tested against an extensive panel of a total of 225 mammalian proteins, including ion channels, G-protein coupled receptors (GPCR), nuclear receptors, kinases, non-kinase enzymes, and drug transporters, cardiac function, and drug-drug interactions. Additionally, these also examined whether Vuti interacted with reported direct targets of glabridin [37], such as estrogen receptor, peroxisome proliferator-activated receptor gamma (PPARγ), and cyclooxygenase 2 (COX-2). Through this off-target screening of an additional 225 targets, there were no significant unexpected targets of Vuti (data not shown). These findings indicate that Vuti does not bind to the reported targets of glabridin other than PON2 and supports the strong in vitro safety of Vuti.

To further investigate the potential neurotoxicity of Vuti in vivo, a standard functional observational battery (FOB) evaluation [47] was performed. Male Sprague-Dawley rats were administered single high doses of Vuti – 500, 1000, and 2000 mg/kg. Subsequently, two observers blindly scored the behavior of animals in the home cage, open field, hand-held, and sensory-motor function at time points of 0.5, 1, 3, 6, and 24 h. Vuti did not induce statistically significant changes in any measured behaviors (Supplemental Table S2), confirming in vivo preclinical safety in terms of neurotoxicity.

## Discussion

In this study, we have elucidated and characterized vutiglabridin, the innovative therapeutic compound that has successfully passed Phase 1 and is currently progressing through Phase 2 trials, as a potential treatment option for PD. The key findings of our research include the following: (1) Vuti efficiently crosses the BBB and is retained in the brain tissue at concentrations approximately 2.5 times greater than in the bloodstream, (2) Vuti binds to PON2 in both its (R) and (S)-enantiomeric forms, (3) Vuti effectively counteracts mitochondrial dysfunction induced by MPP^+^ in neuronal cells through its interaction with PON2, (4) the neuroprotective benefits of Vuti in the MPTP model of PD were significantly diminished in mice with PON2 knockdown, suggesting a critical role for PON2 in its mechanism of action, and (5) its therapeutic efficacy was further evidenced by the preservation of TH + dopaminergic neurons, which was achieved by mitigating astrocytic activation. These findings underscore the potential of Vuti as a novel intervention for PD, offering new avenues for the management of this neurodegenerative disease.

Our study highlights the urgent need for advanced PD therapies, this study points out the potential of targeting mitochondrial dysfunction through novel proteins. Vuti, a clinical-stage compound, has shown significant neuroprotective effects by modulating PON2 in PD models, alongside a successful safety profile in CNS-targeted screenings. Our research also reveals Vuti’s capability to penetrate the BBB, as evidenced by quantitative autoradiography and pharmacokinetic studies, which consistently demonstrated significant brain accumulation. These findings advocate for PON2 as a promising target in PD therapeutic development and support the continued clinical progression of Vuti, despite the limitations of employing whole-body sagittal sections for evaluating brain drug transport.

We constructed a 3D structure of PON2 through homology modeling, providing a valuable foundation for future studies on PON2. Our in silico model revealed that both the (R) and (S)-forms of Vuti bind to PON2. This is an exceptional case where both enantiomers of a compound bind to the same target protein, strengthening the potential of developing Vuti as a racemate. Notably, Vuti showed stronger binding interactions with PON2 compared to glabridin. Structural modifications in Vuti, such as the hydrogenation of double bonds in pyranobenzene and the ethoxification on the resorcinol ring, confer greater conformational flexibility and hydrophobicity [36]. These changes may account for its increased alignment in both hydrogen bonding (ILE57, VAL335) and hydrophobic interaction (PRO59, TYR336) with PON2 (Fig. 4a). However, the molecular docking results do not elucidate the mechanism of how the binding of Vuti increases PON2 protein stability. Further investigations are required to unravel the precise molecular mechanism underlying the PON2 stability.

Our findings reveal that Vuti effectively counteracts MPP^+^-induced mitochondrial dysfunction in neuronal cells by enhancing PON2 protein stability. PON2 possibly interacts with Protein deglycase DJ-1(DJ-1) to promote mitophagy, thereby eliminating damaged mitochondria [48–51]. This suggests that Vuti may have the additional function of interacting with DJ-1 to induce mitophagy to eliminate already damaged mitochondria. Vuti also promoted an increment of TH + neurons in SNpc and striatum, which mitigated the motor impairment in the MPP^+^ -induced PD model. However, in PON2-KD mice, the neuroprotective effects of Vuti were negated, with these mice showing increased susceptibility to MPTP-induced neurotoxicity, evidenced by higher mortality rates, diminished motor behavior, and decreased TH + fiber density, despite no reduction in TH + neurons in the SNpc. These findings emphasize the vital role of PON2 in mediating Vuti’s neuroprotective effects against PD. Additionally, in the AAV-α-syn-A53T model, Vuti not only preserved TH + dopaminergic neurons but also increased DDC + neurons, suggesting its potential to reactivate dormant dopaminergic neurons by reducing astrocytic GABA secretion under inflammatory conditions [15, 17]. Since Vuti inhibits both MAO-A and MAO-B only at concentrations well above those tested, its neuroprotective effects are unlikely to be mediated through MAO inhibition (Supplemental Figure S3). The inclusion of these data is intended to demonstrate that Vuti’s mechanism of action is independent of MAO inhibition, thereby distinguishing it from MAO-B inhibitors such as selegiline or rasagiline. Instead, our primary focus is on the modulation of PON2 activity, which we propose as the key therapeutic mechanism underlying its neuroprotective effects. This study introduces Vuti as a pioneering compound that modulates PON2 for substantial neuroprotection in PD models, setting a foundation for future investigations into its mechanisms and therapeutic applications. The exact mechanism of how Vuti rescues the PD phenotype is unknown. However, it is highly likely that the exact mechanism involves the reduction of ROS by Vuti through the restored activity of PON2. Future work is needed to explore this exciting possibility.

Importantly, while Vuti’s impact on mitochondrial function has been studied in other conditions such as obesity and aging, our study provides the first direct evidence of its disease-specific efficacy in PD models. The loss of neuroprotection in PON2-knockdown mice following MPTP exposure highlights a PD-relevant mechanism, indicating that Vuti’s effects are not merely due to general antioxidative activity. The ongoing Phase 2a clinical trial (NCT06329141), designed to assess the efficacy and safety of Vuti in patients with early-stage PD, is expected to further define its therapeutic potential in humans beyond preclinical models. This mechanistic specificity, combined with Vuti’s favorable safety profile and robust brain penetrance, underscores its promise as a first-in-class, disease-modifying agent for PD. In conclusion, Vuti modulates PON2 specifically and safely as a novel therapeutic strategy and has the potential for groundbreaking advancements in PD treatment.

Our conclusion could also underline the importance of ongoing and future clinical trials to validate these preclinical findings and explore the broader applicability of PON2-targeted therapies in PD and related neurodegenerative disorders.

## Materials and methods

### Study design

The aim of this study was to evaluate the potential neuroprotective effects of Vuti in PD models and to investigate whether such effects are mediated by PON2. SH-SY5Y human neuroblastoma cells, Sprague-Dawley rats, and C57BL/6J mice were used in this study. The QWBA study was performed in SD rats according to an established standard procedure [52] in which plasma pharmacokinetics were assayed first and then sections were obtained from one rat at each time point selected on the basis of plasma pharmacokinetics. In vitro assays were performed with three independent replicates, except for the comprehensive safety panels, which were performed with only two replicates, given the nature of the screening purpose. For animal studies, sample sizes were not calculated through power analysis, but the target was greater than 5 animals per group, except for the pharmacokinetics study where 3 animals per group were considered sufficient due to excessive sacrifice of animals at each time point. For the efficacy studies, drug treatment schemes as well as the behavioral and biological endpoints were previously optimized, in both PD models, the subacute MPTP model and the AAV-α-syn-A53T model [53–55]. Unexpected deaths of PON2-KD animals occurred 5 days after MPTP injection and were excluded from the analysis. Details of randomization, blinding, animal age and sex, and example sample size are noted under each relevant figure and/or method.

### Materials

Vuti was prepared at Glaceum Inc. (Suwon, Korea) according to the protocols from Patent US9783551B2 [56]. Primary antibodies against TH (Cell Signaling Technology, Beverly, MA, USA) and β-actin (Sigma-Aldrich Co., St. Louis, MO, USA) were purchased from commercial sources. Tetramethylrhodamine ethyl ester (TMRE), 5,6-chloromethyl-2’,7’-dichlorodihydro fluorescein diacetate acetyl ester (DCF-DA), and MitoSOX agents were purchased from Molecular Probes (Eugene, OR, USA). All other reagents were purchased from Sigma-Aldrich. Media and culture reagents were products of Gibco Industries Inc. (Auckland, New Zealand). Detailed methods are provided in the Supplemental Materials.

### Quantification of drug concentration in the plasma and brain of mice

Male C57BL/6J mice (8 weeks old, *n* = 24, non-fasted; Charles River Japan (CRJ), OrientBio, Seongnam, Korea) were dosed with 50 mg/kg of Vuti once and sacrificed at 0, 1, 2, 4, 6, 8, 10, 24 h time point (*n* = 3 per time point). Their whole blood was collected via the vena cava in a heparin-coated syringe and their whole brain was collected after perfusion with 0.9% saline. Vuti was quantified in both plasma and brain samples using LC-MS/MS analysis. This study was performed in accordance with the Association for Assessment and Accreditation of Laboratory Animal Care International (AAALAC; Approval No. LCDI-2021-0027).

### Cell culture and treatment

Human SH-SY5Y neuroblastoma cells (ATCC^®^ (CRL-2266™; Manassas, VA, USA) were first cultured in Dulbecco’s Modified Eagle Medium (DMEM)/F12 supplemented with 10% fetal bovine serum (FBS), 100 U/mL penicillin, and 100 µg/mL streptomycin (complete media, CM) at 37 °C with 5% CO_2_. Cells (5 × 10^4^ cells/well) then were cultured in 96-well plates for 24 h followed by incubation in serum-deficient media (SDM, culture media containing 0.5% FBS) for 16 h. Quiescent cells in SDM were incubated with 1 mM MPP^+^ or dimethyl sulfoxide (DMSO) vehicle for 24 h, then treated with Vuti at the designated doses for 24 h. The treated cells were harvested according to the purpose and analyzed.

### PON2 knockdown using shPON2

Lentiviral PON2 shRNA (h) (sc-62838) was purchased from Santa Cruz Co. (Santa Cruz, CA, USA). shRNA lentiviral particle transduction was performed according to the manufacturer’s protocol. Total RNA was isolated with a Trizol reagent (Invitrogen, Carlsbad, CA, USA). Total RNA (1.5 µg) was reverse transcribed using MMLV reverse transcriptase (Promega, Madison, WI, USA), RNasin Ribonuclease inhibitors (Promega), 10 ng of random primers (Invitrogen), and 25 mM of dNTP mix (Gene Craft, Ludinghausen, Germany), according to the manufacturer’s instructions. PON2 knockdown was verified by quantitative PCR, and the primers and methods used are described in the supplementary material.

### Western blot analysis

Cell lysates were prepared on ice in PRO-PREP lysis buffer (10 mM HEPES, pH 7.9, 10 mM KCl, 2 mM MgCl_2_, 0.5 mM dithiothreitol, 1 mM phenylmethylsulfonyl fluoride, 1 µg/mL aprotinin, 1 µg/mL pepstatin A, and 2 µg/mL leupeptin; iNtRON Biotechnology, Gyeonggi-do, Korea). Protein extracts (30 µg) were separated by 12% SDS-PAGE and analyzed by Western blot and an enhanced chemiluminescence system (ECL, Amersham Bioscience, Piscataway, NJ, USA). Anti-PON2 antibody (Santa Cruz Biotech, Dallas, TX, USA) and anti-TH (Pel-Freez Biologicals, Rogers, AR, USA) were diluted at 1:1000. Anti-β-actin antibody (1:3000) was used as a loading control. Band intensities were quantified by densitometry and the ImageJ program (National Institutes of Health, Bethesda, MD, USA).

### MPTP-injected mice and drug administration

Male C57BL/6 mice aged 8–10 weeks with an initial body weight of 20–24 g were purchased from Samtako Bio Korea Co. Ltd (Osan-si, Korea) and PON2-KD mice were generously provided by Dr. Srinivasa T. Reddy (UCLA, CA, USA) [46, 57]. Animals were allowed at least 1 week of acclimatization before being subjected to the study and were housed in a regulated environment with a 12-h/12-h light/dark cycle. Food and water were provided *ad libitum*. The animal experiment was carried out in accordance with the National Institutes of Health Guide for the Care and Use of Laboratory Animals (NIH Publications No. 80–23; revised 1996). All procedures for handling mice were carried out in accordance with “Principles of Laboratory Animal Care” and the Animal Care and Use guidelines of Kyung Hee University, Seoul, Korea (KHSASP-20-163).

MPTP was dissolved in distilled water and injected intraperitoneally for five days from day 10 of the 21-day experiment period. Vuti or rasagiline was administered via oral gavage for 21 days. Vehicles for both Vuti and MPTP (distilled water) were administered to all mice. Mice were randomly divided for all studies. For the first set of studies shown in Fig. 3, each group consisted of 5 animals. For the second set of study shown in Fig. 6, each group consisted of the following animals: (1) wild-type vehicle-only group (Vuti 0; *n* = 5), (2) wild-type Vuti 50 mg/kg group (Vuti 50; *n* = 5), (3) wild-type MPTP + vehicle-only group (Vuti 0; *n* = 8, excluding 2 deaths), (4) wild-type MPTP + Vuti 50 mg/kg group (Vuti 50; *n* = 6), (5) PON2-KD vehicle-only group (Vuti 0; *n* = 7), (6) PON2-KD Vuti 50 mg/kg group (Vuti 50; *n* = 4), (7) PON2-KD MPTP + vehicle group (Vuti 0; *n* = 7, excluding 6 deaths), (8) PON2-KD MPTP + Vuti 50 mg/kg group (Vuti 50; *n* = 8, excluding 5 deaths).

### AAV-α-syn A53T-injected mouse model and drug administration

Male C57BL/6J mice (7 week-age-old) were used for inducing the α-syn A53T model and treating Vuti and vehicle. All mice were maintained in a specific pathogen-free animal facility under a 12:12-h light-dark cycle (lights on at 8:00 AM) at a temperature of 21℃ and allowed *ad libitum* access to food and water [15]. All experiments and animal care were performed in accordance with the guidelines of the Institutional Animal Care and Use Committee (IACUC) of IBS, Daejeon, South Korea (IBS-IBC-2022-12). Mice were anesthetized under isoflurane anesthesia (induction: 3%–4%, maintenance: 1.5%–2%) and placed into stereotaxic (RWD, China). For establishing the α-syn A53T mouse model, 2µL of AAV-CMV-A53T-Asyn or AAV-CMV-EGFP (5 × 10^12^ genomic copies/mL, packaged by the IBS virus facility) was injected into the SNpc (AP − 3.2 mm, ML ± 1.3 mm, DV − 4.0 mm relative to the bregma; 0.2µL/min) bilaterally. Oral administration of 50 mg/kg Vuti (Vuti 50) or the vehicle (Veh) was started 5 weeks after the virus injection, once a day for 12 weeks. Mice were used for behavior tests and immunostaining after completing drug treatment. Vertical grid test was performed as described before [15, 58]. For 5 days, mice were allowed for habituation in the test room. Prior to the trial session, the mice were acclimated to the vertical grid for 2 days. For the experiment, individual mice were gently placed 3 cm from the top, facing upward, and allowed to turn around and climb down. The trial was repeated whenever the mouse failed to climb down within 60 s. Starting from the fourth week after the injection, trials are conducted at two-week intervals.

### Quantitative whole-body autoradiography (QWBA) assay in rat

Male 8-week-old Sprague Dawley rats (*n* = 5, 16 h fasted; Charles River Laboratories Japan Inc., Yokohama, Japan) were orally administered 10 mg/kg of ^14^C-Vuti (equivalent to radioactivity of 3.7 MBq/kg, Glaceum Inc., Suwon, Korea). After 2, 6, and 24 h, each rat was euthanized by CO_2_ inhalation. The nasal cavity and anus were filled with 4% w/v sodium carboxymethyl cellulose, and the carcass was frozen in a dry ice-acetone mixture. Whole-body sagittal sections of 30 μm thickness were cut with a cryo-microtome (CM3600, Leica Biosystems, Wetzlar, Germany), freeze-dried, covered with 4-µm thick protective membrane (Diafoil, Mitsubishi Plastics, Tokyo, Japan), and then exposed to the imaging plate (TYPE BAS SR2040, Fujifilm, Tokyo, Japan) for 24 h in a sealed lead box together with the plastic standard samples (CFQ7601, Amersham Biosciences Corp., NJ, USA) for calibration. The radioactivity was measured by the bio-imaging analyzer system (FUJIX-BAS2500, Fujifilm, Tokyo, Japan; resolution µm, gradation 256, sensitivity 10000, latitude 4), in which the radioactivity in organs was converted to the photo-stimulated luminescence per unit area (PSL/mm^2^) minus the background radioactivity of the plastic standard samples. Tissue radioactivity in whole-body auto-radiograms was quantified by densitometry using an MCID image analysis software (v. 7.0, MCID Image Analysis Software Solutions for Life Sciences, Cambridge, UK). Radioactivity concentrations were expressed as ng equivalents of ^14^C-Vuti per gram tissue (ng eq/g tissue). The study protocol referenced numerous established methods for QWBA [52, 59, 60] and was reviewed and approved by the Institutional Animal Care and Use Committee (IACUC: 2017-016).

### Octanol-water partition coefficient measurement

1000 µg/mL of Vuti was mixed with octanol, and an aliquot of 2 mL was mixed with the same amount of aliquot of 2 mL of pH7.4 phosphate buffer. The mixed solution was shaken at room temperature for 24 h, and the aqueous and octanol phase was separately analyzed for Vuti concentration via HLPC. The limit of quantification (LOQ) was 0.2012 µg/ml. Three replicates were performed. Because no concentration was detected in the aqueous phase, LOQ value was used; the actual concentration in the aqueous phase is expected to be lower than LOQ. The octanol-water partition coefficient Log D was calculated as log of concentration in octanol phase divided by concentration in aqueous phase.

### P-glycoprotein (P-gp) efflux assay

P-gp efflux ratio was determined in MDCKII-hMDR1, MDCKII [61], and Caco-2 cell [62] monolayer via LC-MS/MS analysis, in accordance with the standard FDA guideline on transporter-mediated drug interactions. 10 µM of GF120918 was used as P-gp inhibitor. The apical (A-to-B) and basolateral (B-to-A) permeability coefficient (P_app_) were measured to yield the efflux ratio.

### Plasma concentration of ^14^C-labelled Vuti

For measuring the radioactive concentration in the plasma, blood (250 uL) was collected from the tail vein of the animals (*n* = 3) of the exact same conditions as the animals used for QWBA assay. The plasma was separated by centrifugation (8000xg, 4℃, 5 min) into a scintillation vial, dissolved with 2 mL of tissue solubilizer (Soluene-350, PerkinElmer Inc., MA, USA), and mixed with 10 mL of scintillator (Hionic-Fluor, PerkinElmer Inc., MA, USA). The radioactivity (ng eq./mL) was measured using liquid scintillation counter (1900CA, 2500TR, 2700TR, PerkinElmer Inc., MA, USA) for 2 min, calibrated with the scintillator as the background sample. Then the tissue to blood ratio was calculated. The protocol was reviewed and approved by the Institutional Animal Care and Use Committee.

### LC-MS/MS analysis

LC-MS/MS analysis to quantify Vuti used previously set conditions [51]. Briefly, HPLC-ESI-MS/MS analyses were performed using a 1200 series HPLC system (Agilent Technologies, Wilmington, DE) coupled to 6490 Accurate-Mass Triple Quadrupole Mass Spectrometer (Agilent Technologies, Wilmington, DE). Mass detection was performed in the positive ion mode, and the column temperature was maintained at 40 °C using a thermostatically controlled column oven. The column used for the separation was Hypersil GOLD C18 column (2.1 × 100 mm ID; 1.9 μm, Thermo Science). The mobile phases consisted of 0.1% acetic acid in distilled water (solvent A) and 0.1% acetic acid in ACN: MeOH = 3:1 (v/v) (solvent B). The LC gradient was as follows: 0–3 min, B 20%; 3–5 min, B 20 − 80%; 5–8 min, B 80%; 8–10 min, B 80 − 20%; and 10–15 min, B 20%. The flow rate was 250 µL/ min, and the total run time was 15 min. For multiple reaction monitoring (MRM) analyses, the target transition used was 355.1/151.0 m/z for Vuti. The collision energy was 17 V and nitrogen was used as the desolvation gas at a flow rate of 11 L/min and at 300 °C. Skyline software (MacCoss Laboratory, University of Washington, Seattle, WA) was used to process the LC-MS/MS data.

### Cloning, expression, and purification of recombinant HsPON2

The codon-optimization of *Hs*PON2 was for *Escherichia coli* system and synthesized according to the full-length open reading frame (GenBank accession No. AAC41995.1) from National Center for Biotechnology Information (NCBI; http://www.ncbi.nlm.nih.gov). The *Hs*PON2 gene was amplified by a standard PCR method with a primer set (Forward; 5’-GCACTC*CATATG*GGGGCATGGGTCGGGTGTGGGTTGGCCGGGGATAGAGCCGGCTTTC-3’, Reverse; 5’-GCACTC*CTCGAG*AAGCTCGCAGTACAATGC-3’) and inserted into an expression vector (pET21b; New England Biolabs, USA) using *Nde*I and *Xho*I restriction enzyme sites (underlined). Notably, a six-His tag was fused to C-terminus of the protein to facilitate protein purification. The recombinant construct was transformed into *E. coli* BL21 (DE3) competent cells. The transformed cells were activated overnight and transferred to 1 L of LB broth (BD bioscience, USA) with 100 µg/mL ampicillin (Duchefa Biochemie, The Netherlands) containing 0.1% glucose, and then incubated at 37 ℃ and 200 rpm in incubator (Vision Scientific, Korea) until reaching the optical density of 0.6–0.8 at 600 nm (OD_600_). The overexpression of the recombinant *Hs*PON2 protein was induced by the addition of 0.5 mM isopropyl β-D-1-thiogalactopyranoside (IPTG) (Duchefa Biochemie), and then further incubated for 7 h at 25℃ and 170 rpm. After the protein expression, the cells were harvested and resuspended in 30 mL of lysis buffer (10 mM Bicine, pH 9.0, 50 mM NaCl) and lysed for 4 min by sonication (30% Amplitude, 4 s sonication and 6 s rest) (Sonics and materials, USA). To obtain *Hs*PON2 forming inclusion bodies, the supernatant was removed by centrifugation (12,000 rpm, 4℃) for 20 min. The pellet was resuspended in 30 mL of UREA buffer (10 mM Bicine, pH 9.0, 50 mM NaCl, 4 M UREA). After that, the cellular debris was removed by centrifugation (12,000 rpm, 4℃) for 20 min. The supernatant was filtered by the 0.45 μm syringe filter (GVS, Italy). The protein refolding was conducted by the dialysis with buffer of 2 L (10 mM Bicine, pH 9.0, 50 mM NaCl, 1 mM CaCl_2_). The refolded *Hs*PON2 was purified using affinity chromatography with a 10 mL of Ni-NTA resin (QIAGEN, Germany) and an elution buffer (10 mM Bicine, pH 9.0, 50 mM NaCl, 250 mM Imidazole). The eluted *Hs*PON2 protein was analyzed by a 12% SDS-PAGE and concentrated up to approximately 1 mL by 10 kDa filter-size amicon (Merck Millipore, USA). For activity assay of *Hs*PON2, the sample buffer was changed into activity buffer (20 mM Bicine, pH 8.0, 1 mM CaCl_2_). Subsequently, the purified *Hs*PON2 was confirmed by using the western-blot assay with the anti-histidine antibody (MBL, England).

### PON2 3D modeling and molecular docking study

The 3D structure of PON2 was created by prediction using homology modeling of its family protein PON1, whose 3D structure is known and which shares 61.7% sequence identity and 79.2% sequence similarity. The structure of PON1 (PDB ID: 1V04) was obtained from the RCSB Protein Data Bank (http://www.rcsb.org). The 3D structure of PON2 was predicted using Discovery Studio (DS) 2018 (BIOVIA, San Diego, CA, USA). The structural stability of the constructed model of the PON2 protein was verified by molecular dynamics simulation, which was run for a total of 10 ns, and Root-mean-square Deviation (RMSD) and Root-mean-square Fluctuation (RMSF) were investigated to determine the system stability. Phi (Φ) and psi (Ψ) torsion angles and interatomic collisions were reviewed for all amino acids by Ramachandran plot. Molecular docking calculations were performed using Genetic Optimization for Ligand Docking (GOLD v5.2.2, The Cambridge Crystallographic Data Centre, Cambridge, UK), an automated docking program that predicts the binding mode of the ligand by genetic algorithm [63, 64]. The geometry of the compounds – (R) and (S) forms of Vuti and glabridin – was optimized by energy minimization using Minimize Ligands tool in DS. All the active site residues within 10 Å radius sphere of the center were included for the calculation. The number of docking runs was set to 50 for each compound. All other parameters were set to as default values.

### Cellular thermal shift assay (CESTA^®^) of PON2 in brain tissue

Interaction of PON2 and Vuti in mouse brain tissue was evaluated by Cellular Thermal Shift Assay (CESTA^®^) in vivo [65] (Pelago Bioscience AB, Solna, Sweden). Briefly, mice were orally administered vehicle or Vuti at 50 mg/kg (Vuti 50) or 100 mg/kg (Vuti 100) for 5 days. Brain tissue pieces were pulverized under frozen conditions in a mortar with liquid nitrogen. Equal amounts of tissue powder were placed into 8 or 12 separate PCR strip tubes for each set of the treated tissues and kept frozen until heat challenged. Pre-heated 60 µl HBSS buffer was added to the tubes and samples were immediately subjected to an 8 or 12-step heat challenge for 3 min between 37 °C and 59 °C. Samples were then snap-frozen in liquid nitrogen and lysed by freeze-thawing. After the first round of freeze thawing 1% Brij 35 was added to the samples and the samples were incubated with shaking for 20 min at 4 °C before the two last rounds of freeze thawing. After centrifugation at 20 000 x g for 20 min, 35 µl of the supernatant was mixed with 17,5 µl gel loading buffer (NuPAGE LDS sample buffer, Thermo Fisher Scientific, Waltham, MA, USA). Total protein lysate (6 µg) was separated on 12% SDS-PAGE gels. Each sample was run on two separate gels for technical duplicates. Western Blot detected PON2 protein (abcam, ab183718) normalized to SOD-1, a temperature stable protein band observed in the full protein gel detection with house-keeping gene.

### Mass spectrometry-based binding assay

The method followed the concept of previously established protocol [41]. Briefly, 10 nM Vuti was incubated with various concentrations of PON2 (0, 5, 10, 20, 50, 100, 200, and 500 nM) diluted with buffer (10 mM, pH 7.6, 50 mM, CaCl_2_) at 30 °C for 30 min. rePON2 was separated using 7 K size-exclusion chromatography column (Thermo Fisher Scientific, Waltham, MA, USA) and was incubated with a denaturing buffer (1% formic acid in acetonitrile) at 80 °C for 10 min. The supernatant, which now contains rePON2-bound Vuti, was collected, dried, and re-suspended in acetonitrile, and the drug quantification was performed via LC-MS/MS analysis.

### Cell-based mitochondrial activity assays

A systemic cell-based mitochondrial function analysis system was established using SH-SY5Y cells in 96-well plates based on fluorescence detection methods as described previously [55, 66]. The assay system covers quantitative assays for methyl thiazyl tetrazolium-mitochondrial dehydrogenase activity (MTT), TMRE-based mitochondrial membrane potential (TMRE), ATP contents, and ROS of cells. Cells in black 96-well culture plate were incubated with 200 nM TMRE and 0.5 µM Hoechst 33,342 for 30 min at 37 °C in phenol red-free SDM, or with 1 µM CM-H_2_DCFDA or 5 µM MitoSOX and 0.5 µM Hoechst 33,342 for 1 h at 37 °C. Fluorescence intensities at 550 nm/580 nm for TMRE, at 510 nm/580 nm MitoSOX, or at 494 nm/522 nm for DCF-DA were normalized by Hoechst intensity at 355 nm/480 nm (Spectramax Gemini EM, Molecular Devices, Sunnyvale, CA, USA).

Endogenous OCR was measured in adherent SH-SY5Y cells using a Seahorse XF-24 Analyzer (Seahorse Bioscience, Billerica, MA, USA) following the manufacturer’s protocol with minor modifications [67]. Briefly, OCR assays were performed using SH-SY5Y cells (5.0 × 10^3^ cells/well) equilibrated in DMEM without sodium bicarbonate in XF-24 microplates. After measuring basal OCR for 3 min, oligomycin (1 µg/mL), carbonyl cyanide-p-trifluoromethoxyphenylhydrazone (FCCP) (0.3 µM), and rotenone (0.1 µM) were consecutively added into each well to reach their final working concentrations. The OCR was calculated from 3-min measurement cycles and normalized to cell number.

### PON2 activity assay

To assess PON2 activity following Vuti treatment, we infected a primary cultured cortical astrocytes with AAV-CMV-αsyn-A53T virus. Five days post-infection, we treated the cells with Vuti for 24 h and conducted esterase and lactonase assays. For the esterase activity evaluation, reactions were initiated by adding 1 mM p-nitrophenyl acetate (pNPA) to the PON2 activity assay buffer. Enzymatic hydrolysis was monitored by measuring the absorbance at 412 nm. For lactonase activity, the reactions were initiated with 1 mM thio-butyrolactone (TBBL) and 1 mM 5,5’-dithiobis(2-nitrobenzoic acid) (DTNB) in the PON2 activity assay buffer, with absorbance measured at 420 nm to monitor enzymatic hydrolysis.

### PON2 knockdown using shPON2

Real-time quantitative RT-PCR (qRT-PCR) was performed using primers for human PON2 (5’- CCA AGC AAG GGA CAG AAA AG -3’ and 5’- TCA CAG TGC CAG AAG TGA GG -3’) and 18S rRNA (5’-GAG CGA AAG CAT TTG CCA AG-3’ and 5’-GGC ATC GTT TAT GGT CGG AA-3’) on a Roter-Gene Q (Qiagen, Hilden, Germany) with 2x AmpiGene^®^ qPCR Green Mix Lo-ROX (Enzo Biochem, NY) at 95 °C for 10 min, followed by 45 cycles of 95 °C for 5 s and 60 °C for 15 s and 72 °C for 20 s. Measurements were performed in duplicate for each sample. The quantity of mRNA was corrected by simultaneous measurement of nuclear DNA encoding 18 S rRNA. The relative quantification in gene expression was determined using the 2-ΔΔCt method. Relative levels of mRNA expression are presented as fold changes compared to those under the control condition.

### Measurement of intracellular H_2_O_2_ levels in astrocytes

Intracellular H_2_O_2_ levels in astrocytes were measured using a genetically encoded oROS-G plasmid, which is highly sensitive and specific for H_2_O_2_ with rapid on-and-off kinetics [44]. The plasmid was generously provided by Dr. Andre Berndt and was cloned under the GFAP104 promoter. Transfection into primary cultured hippocampal astrocytes was carried out using the NeoNTM transfection system (Invitrogen). Astrocytes were seeded into 96-well black plates (ibidi, USA) and treated with 6-OHDA (10 µM) in the presence of 1, 10, and 30 µM of Vuti. Fluorescence changes were monitored using a Nikon A1 confocal microscope over a period of 21 h for live cell imaging. The fluorescence intensity for each region of interest (ROI) was analyzed using Nikon software, and the data were normalized to control values for the IC_50_. The IC_50_ value for Vuti was determined using GraphPad Prism software.

### MAO-A and MAO-B enzyme assay

#### Horseradish peroxidase (HRP) /Amplex red (AR) based MAO-A and MAO-B enzyme activity assay

MAO-A or MAO-B enzyme solutions were prepared by diluting 10 µL of a 2.5 mg/mL MAO-A or MAO-B stock into 10 mL of 50 mM sodium phosphate buffer (pH 7.4). A substrate assay mixture was then prepared by combining benzylamine (for MAO-B) or tyramine (for MAO-A) from a 100 mM stock (200 µL in 10 mL phosphate buffer, final concentration 1 mM) with 100 µL of a 20 mM AR reagent stock (final concentration 0.1 mM) and 100 µL of a 200 U/mL HRP stock (final concentration 1 U/mL) in 10 mL phosphate buffer. In each well, 49 µL of the enzyme solution (final enzyme concentration approximately 2.5 µM) was dispensed, followed by 1 µL of the test compound. Next, 50 µL of the substrate assay mixture was added, and the reaction was carried out at 37 °C for 60 min. The production of resorufin was quantified by fluorescence measurement (excitation at 540 nm and emission at 580 nm).

#### ROS Glo^™^ based MAO-A and MAO-B enzyme activity assay

An enzyme solution was prepared by diluting 10 µL of a 2.5 mg/mL MAO-A or MAO-B stock in 10 mL of 50 mM sodium phosphate buffer (pH 7.4). A substrate solution was prepared by diluting 200 µL of a 100 mM benzylamine (or tyramine) stock in 10 mL phosphate buffer to achieve a final concentration of 1 mM. For each 96-well plate experiment, 12.5 µL of a 1× H_2_O_2_ probe solution was added to 1 mL of the probe dilution buffer from the ROS Glo^™^ kit. Separately, 40 µL of cysteine and enhancer were diluted into 4 mL of Luciferin solution. In each well, 49 µL of the enzyme solution was dispensed, followed by 1 µL of the test compound, and incubated for 30 min. Then, 49 µL of the substrate solution was added, and the reaction proceeded at 37 °C for 60 min. Next, 10 µL of the H_2_O_2_ probe solution was added and incubated for 15 min. Subsequently, 40 µL of a luciferin solution containing cysteine and an enhancer was added, followed by an additional 30-minute incubation at 37 °C. Luminescence was subsequently recorded using a microplate luminescence reader (SpectraMax i3, Molecular Devices).

### Animal behavior test

To determine forelimb and hindlimb motor coordination and balance, we performed the rotarod test as described previously [68, 69]. Also, the pole test was performed to measure bradykinesia. For the rotarod test, the animals were pre-trained on the rotating bar of a rotarod unit set (LE 8500, Letica, Spain) on days 18 and 19 before the test on day 20. During pre-training, three trials per day were performed (5 rpm rotation speed on the first day, 15 rpm rotation speed on the second day). Mice were kept on the rotating bar (7.3-cm diameter) for 5 min on each trial. On day 20, the time spent on the rotating bar at 20 rpm, which was defined as the latent period, was recorded. Performance was recorded as 300 s if the latent period exceeded 300 s. Mice had at least 5 min of rest between trials to reduce stress and fatigue. Each animal underwent three test trials, and the mean of the test results was subjected to statistical analysis. For the pole test, mice were held on the top of the pole (diameter 8 mm, height 55 cm, with a rough surface). The time to land down and place four fee on the floor was recorded as the time for locomotion activity (T-LA).

### Immunohistochemistry of TH-positive neurons

On day 21, mice were anesthetized with sodium pentobarbital (50 mg/kg intraperitoneally) and transcardially perfused with saline containing 0.5% sodium nitrate and heparin (10 U/mL) and then fixed with 4% PFA in 0.1 M phosphate-buffer (PB, pH 7.2). Dissected brains were post-fixed overnight in buffered 4% PFA at 4 °C and stored in a 30% sucrose solution until they sank. Brains were cryosectioned into 30 μm thick coronal sections in a Cryostat (Microsystems AG, Leica, Wetzlar, Germany) and stored until use at 4℃ in a cryoprotectant (25% ethylene glycol, 25% glycerol, 0.2 M PB, and water). All sections were collected in six separate series and processed for immunostaining.

Free-floating brain sections were processed with the avidin-biotin peroxidase complex (ABC) complex (Vector Laboratories, Burlingame, CA, USA) method [70]. Briefly, brain sections were incubated with primary rabbit anti-TH antibodies (1:1000; Pel-Freez Biologicals, Rogers, AR, USA) overnight at 4 °C, with biotinylated anti-rabbit IgG (1:400) for 1 h at room temperature, and then with the ABC solution (1:1:200) for 1 h at room temperature. The immunoperoxidase signals of TH was detected by incubating sections with 0.5 mg/ml 3,3′-diaminobenzidine (Sigma, St. Louis, MO, USA) in 0.1 M PB containing 0.003% H_2_O_2_. Stained brain sections were mounted on gelatin-coated slides and imaged under a bright-field microscope (Olympus Optical, Tokyo, Japan). TH-positivity in the ST was measured by the optical density of TH-positive fibers at x40 magnification using ImageJ software (National Institutes of Health, Bethesda, MD, USA). TH-positive cells in the SNpc in brain tissue Sect. (5 sections/series) were counted at x100 magnification as previously described [70, 71]. The sections used for counting covered the entire SN, from the rostral tip of the pars compacta (SNpc) to the caudal end of the pars reticulata (SNr) (anteroposterior, -2.06 to -4.16 mm from the bregma). The SN area was delineated on a x1.25 objective, resulting in a counting grid of 150 × 150 μm. The estimate of the total number of neurons was calculated according to the Optical Fractionator Equation.

### Immunohistochemistry and fluorescence imaging

Mice were deeply anaesthetized with isoflurane and perfused transcranial with 0.9% saline ice-cold 4% paraformaldehyde (PFA). Excised brains were postfixed at 4 °C overnight and transferred to 30% sucrose for 48 h for dehydration. Coronal sections of 30 μm thickness (20 μm thickness for super-resolution imaging) were prepared in a cryostat and stored in a storage solution at 4 °C. Brain tissues were washed in PBS and incubated in a blocking solution (0.3% Triton X-100, 2% Donkey Serum, 2% Goat Serum in 0.1 M PBS). Primary antibodies were added to the blocking solution at desired dilution and the sections were incubated in a shaker at 4 °C overnight. Primary antibodies used for immunostaining were anti-TH (rabbit, 1:200, Pel-freez, P40101-150), GFAP (chicken, 1:500, Millipore, AB5541), Iba-1 (rabbit, 1:500, Wako, 019-19741), DDC (rabbit, 1:200, abCam, ab3905), GABA (guinea pig, 1:200, Millipore, AB175), anti-PON2 (rabbit, 1:200, abCam, ab183710). Unbound antibodies were washed off using PBS, followed by corresponding secondary antibody incubation (in blocking solution) for 1–2 h at room temperature. Unbound antibodies were washed with PBS and DAPI was added to PBS (1:1500 dilution). Sections were mounted with fluorescent mounting medium (Dako) and dried. A series of fluorescent images were obtained using Zeiss LSM900 confocal microscope. Super-resolution image for PON2 signals imaged by Zeiss Lattice SIM (Structure-illumination microscope) and all images were processed using ImageJ (Fiji) software.

### Statistical analysis

Results are presented as mean ± standard error of the mean (SEM). Statistical significance between experimental groups was evaluated by one-way ANOVA with Tukey post-hoc analysis using GraphPad Prism (version 9.4.0, GraphPad Software, San Diego, CA, USA) unless otherwise indicated. Values of *p* < 0.05 were considered statistically significant.

## Supplementary Information

Below is the link to the electronic supplementary material.

Supplementary Material 1

## Data Availability

All data associated with this study are present in the main text or the supplementary materials. Information on the 3D structure of PON2 can be obtained by contacting Keun Woo Lee at kwlee@gnu.ac.kr. Vuti can be obtained through a material transfer agreement from Glaceum by contacting Hyung Soon Park at hspark@glaceum.com.

## Abbreviations

PD: Parkinson’s disease
α-syn: α -synuclein
PON2: Paraoxonase 2
BBB: Blood-brain barrier
TH+/DDC+: Tyrosine hydroxylase positive neurons /dopa decarboxylase positive neurons
ROS: Reducing reactive oxygen species
SNpc: Substantia nigra pars compacta
OXPHOS: Oxidative phosphorylation
MAO-B: Monoamine oxidase B
GABA: Gamma-aminobutyric acid
MPTP: 1-methyl-4-phenyl-1,2,3,6-tetrahydropyridine
Vuti: Vutiglabridin
P-gp: P-glycoprotein
QWBA: Quantitative whole-body autoradiography
AUC: Total area under the curve
OCR: Oxygen consumption rate
GlycoATP: ATP production rates through glycolysis
mitoATP: ATP production rates through mitochondria
3D: Three-dimensional
GOLD: Genetic Optimization for Ligand Docking
KD: Knockdown
shPON2: PON2 shRNA
PON2-KD: PON2 knockdown
Vuti 100: 100 mg/kg Vuti
Vuti 50: 50 mg/kg Vuti
AAV: Adeno-associated virus
AAV-A53T: AAV-α-syn mutant
A53T group: AAV-A53T model
CNS: Central nervous system
GPCR: G-protein coupled receptors
PPARγ: Peroxisome proliferator-activated receptor gamma
COX-2: Cyclooxygenase 2
FOB: Functional observational battery
DJ-1: Protein deglycase DJ-1
TMRE: Tetramethylrhodamine ethyl ester
DCF-DA: 5,6-chloromethyl-2’,7’-dichlorodihydro fluorescein diacetate acetyl ester
AAALAC: Association for Assessment and Accreditation of Laboratory Animal Care International
DMEM: Dulbecco’s Modified Eagle Medium
SDM: Serum-deficient media
DMSO: Dimethyl sulfoxide
SEM: Standard error of the mean
