# Supplementary material for: Neurotherapeutic effects of Vutiglabridin as a Paraoxonase-2 modulator in preclinical models of Parkinson’s disease
Source: Mol Neurodegener. 2025 Oct 17;20:110. doi: 10.1186/s13024-025-00896-z (PMC12535076; doi:10.1186/s13024-025-00896-z)
Supplement: Supplementary file 1 — Supplementary Material 1 [file 13024_2025_896_MOESM1_ESM.docx]

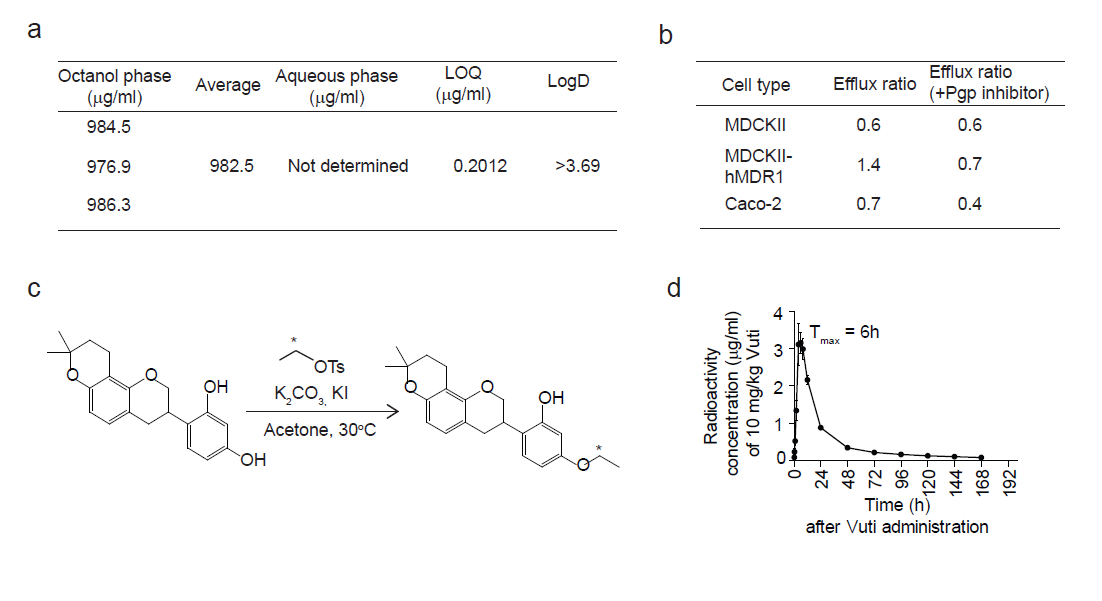


**Supplemental Figure S1. Properties of Vuti and 14C-labeled Vuti**

(a) Measurement of Vuti concentration in octanol and aqueous phase (pH7.4 phosphate buffer) via HPLC. The mean value of three replicates was used, and the limit of quantification (LOQ) value was used to calculate Log D as a log of concentration in the octanol phase divided by the concentration in the aqueous phase. (b) Efflux ratios (Papp(B→A)/Papp(A→B)) of Vuti in three types of cell monolayers, with or without P-glycoprotein (P-gp) inhibitor GF120918. (c) Schematic of 14C-labeling of Vuti. Attachment to the ethoxy group is warranted because it is robustly maintained throughout metabolism. (d) Plasma radioactivity concentration in Sprague-Dawley rats after a single administration of 10 mg/kg of 14C-Vuti (n = 3).


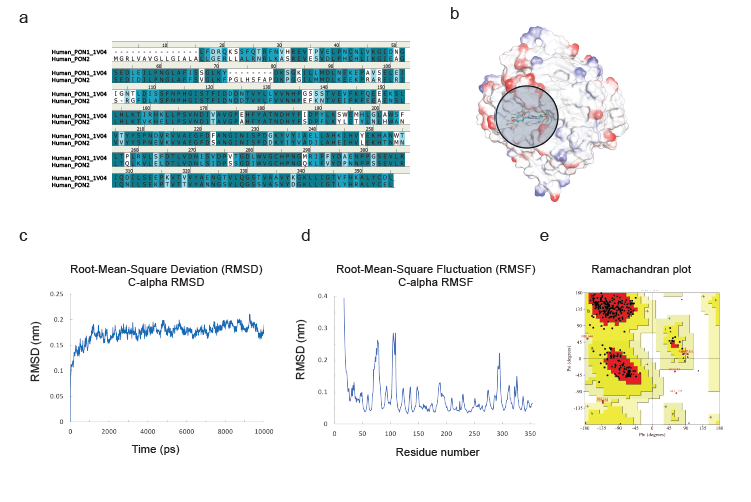


**Supplemental Figure S2. De novo 3D structure of PON2 and binding interaction with Vuti.**

The De novo 3D structure of PON2 was constructed via homology modeling to PON1 and binding interaction with Vuti was assessed. (a) Sequence comparison of PON1 and PON2 proteins shows sequence identity of 61.7% and sequence similarity of 79.2%. (b) De novo 3D protein structure of PON2 and the docking site for the tested compounds. (c, d) Molecular dynamic simulation showing RMSD (c) and RMSF (d) graphs for 10 ns show stable maintenance and no significant movement, except for the amino acid residues forming the loop with the end of the N-terminus. (e) Ramachandran plot showing phi(Φ) and psi(Ψ) torsion angles and interatomic collisions. 86.6% of torsion angles were located in the normal distribution area and most amino acids are within the acceptable range.


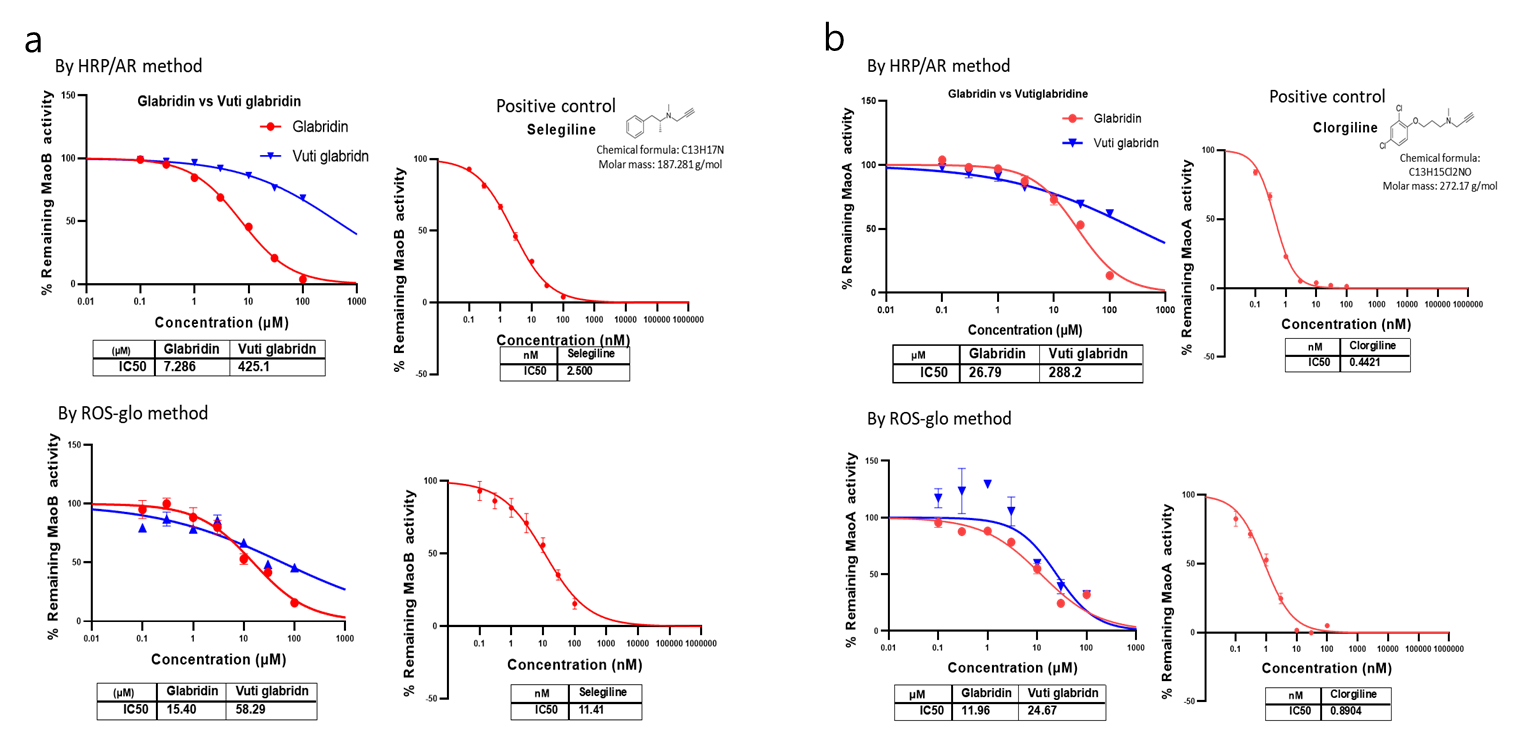


**Supplemental Figure S3. MAO-A and MAO-B inhibition by Vuti by Glabridin and Vutiglabridin**

Enzyme assays were performed to evaluate the inhibitory effects of glabridin and vutiglabridin. (a) In the MAO-B assay using the HRP/AR method, glabridin and vutiglabridin exhibited IC_50_ values of 7.29 μM and 425.10 μM, respectively, while the positive control, selegiline, showed an IC_50_ of 2.5 nM. In a separate MAO-B assay using the ROS-Glo method, glabridin and vutiglabridin yielded IC_50_ values of 15.40 μM and 58.29 μM, respectively, compared to 11.41 nM for selegiline. (b) In the MAO-A assay using the HRP/AR method, glabridin and vutiglabridin exhibited IC_50_ values of 26.79 μM and 288.20 μM, respectively, while the positive control, clorgiline, showed an IC_50_ of 0.44 nM. In a separate MAO-B assay using the ROS-Glo method, glabridin and vutiglabridin yielded IC_50_ values of 11.96 μM and 24.67 μM, respectively, compared to 0.89 nM for clorgiline.


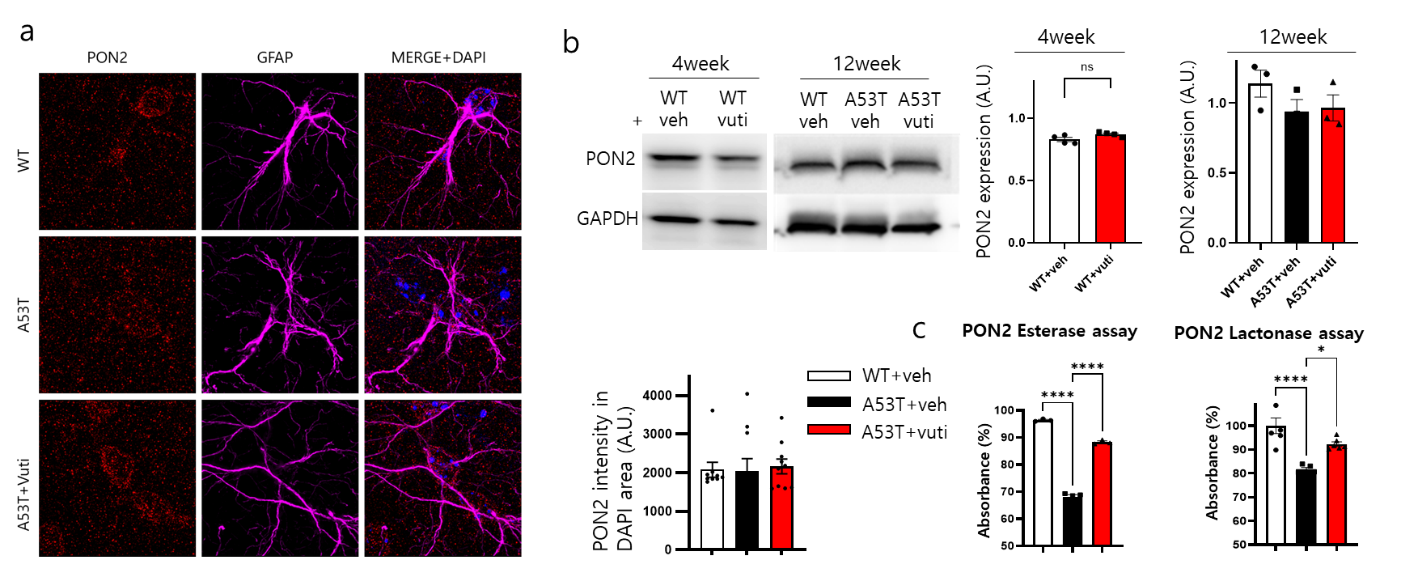


**Supplemental Figure S4. PON2 expression level and activity change after treating Vuti.**

PON2 protein expression level and enzyme activity were confirmed. (a) Immunohistochemical analysis was performed using antibodies against PON2 and GFAP in WT + veh, A53T + veh, and A53T + vuti groups. Super-resolution images were acquired and analyzed with ImageJ to quantify PON2 intensity within DAPI-stained astrocytic regions. (b) Western blot analysis of PON2 protein levels in mouse brain tissues following vutiglabridin treatment. Left, representative blots of PON2 and GAPDH (loading control). Right, quantification of normalized PON2 expression levels. In the 4-week treatment group, the midbrain of WT + veh and WT + Vuti were compared. In the 12-week treatment group, the SNpc tissue of WT + veh, A53T + veh, and A53T + vuti were compared. (c) Enzymatic activity was evaluated via PON2 esterase and lactonase assays in primary cultured cortical astrocytes. *, p < 0.05; **, p < 0.01; ***, p < 0.001; vs WT + veh. #, p < 0.05; ##, p < 0.01 vs A53T + veh. p-values are from a one-way ANOVA followed by Tukey’s test.


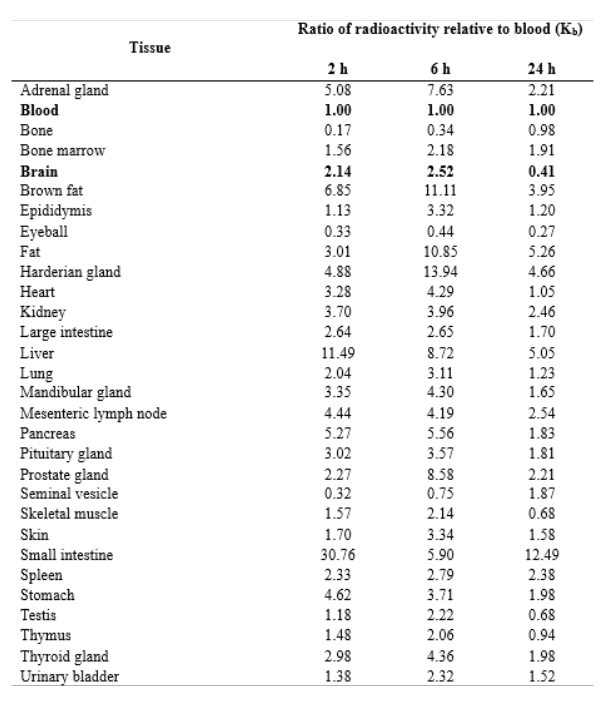


**Supplemental Table S1. Distribution of radioactive-labeled Vuti in Sprague-Dawley rat**

Male Sprague Dawley rats of 8 weeks of age were orally administered 10 mg/kg of 14C-labeled Vuti and its distribution throughout the whole body was quantified by measuring radioactivity on the tissues of the whole-body sagittal sections made at 2, 6, and 24 h after the drug administration, and was normalized to the radioactivity in blood (Kb).


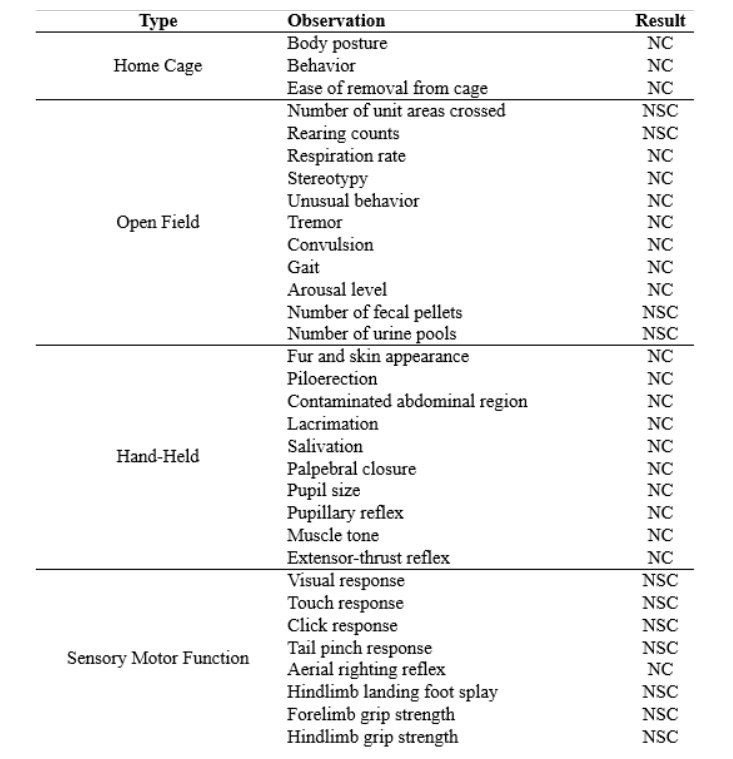


**Supplemental Table S2. Functional observation battery (FOB) evaluation**

Male 6-week-old Sprague-Dawley Rat was orally administered with 0, 500, 1000, or 2000 mg/kg of Vuti (n = 8 per group). Two observers performed blind observations following the standard guideline (3) at 0.5, 1-, 3-, 6-, and 24-hours post-dose on the basis of the pharmacokinetics parameters. No statistically significant or biologically relevant differences in all parameters were observed for all dose groups at all time points. NC = No change, NSC = No statistically significant change.
